# Detection Strategies for HER2 and miR-21 in Breast Cancer: Current Approaches and Future Perspectives- Hybrid Systematic Review and Bibliometric Mapping

**DOI:** 10.3390/diagnostics16193179

**Published:** 2026-09-30

**Authors:** Diana-Maria Burța, Simona Daniela Cavalu, Luminita Fritea, Tunde Horvath, Sebastian Nemeth, Ioana Aura Istoan, Florin Banica

**Affiliations:** 1 Doctoral School of Biological and Biomedical Sciences, University of Oradea, 1 University Street, 410087 Oradea, Romania; burta.dianamaria@student.uoradea.ro; 2 Department of Preclinical Sciences, Faculty of Medicine and Pharmacy, University of Oradea, Pta 1 Decembrie, 410087 Oradea, Romania; 3 Department of Pharmacy, Faculty of Medicine and Pharmacy, University of Oradea, 29 N. Jiga Street, 410087 Oradea, Romania; thorvath@uoradea.ro (T.H.); snemeth@uoradea.ro (S.N.); fbanica@uoradea.ro (F.B.); 4 Faculty of Medicine and Pharmacy, University of Oradea, 410087 Oradea, Romania; istoan.auraioana@student.uoradea.ro

**Keywords:** HER2, miR-21, breast cancer, molecular biomarkers, liquid biopsy, biosensors, precision oncology

## Abstract

**Background/Objectives:** Breast cancer is still one of the main causes of mortality and morbidity at a global level, highlighting the need to identify robust biomarkers for early diagnosis, prognostic stratification and optimization of therapeutic decisions. Among these biomarkers, HER2 (Human Epidermal Growth Factor Receptor 2) and miR-21 represent the complementary levels of tumor biology, reflecting oncogenic signaling at the protein level and post-transcriptional regulation of gene expression. This hybrid systematic review and bibliometric mapping aims to critically analyze current methods for determining HER2 and miR-21 biomarkers, highlighting their advantages, limitations and clinical applicability, as well as future development directions in the field of precision oncology diagnostics. **Methods:** The bibliometric analyses were performed to investigate the intellectual structure, research trends, and collaboration patterns within the field of HER2 and miR-21 detection in breast cancer. A PRISMA-guided literature search was conducted in the Web of Science Core Collection (WoSCC), PubMed, and Scopus. After duplicate removal, screening, and eligibility assessment, 305 publications were included in the final review corpus. Bibliometric analyses of these 305 publications were performed using VOSviewer to investigate publication output, keyword co-occurrence, co-authorship, and institutional and country collaboration patterns. **Results:** Bibliometric analyses revealed the intellectual structure, research trends, and collaboration patterns within the field of HER2 and miR-21 detection in breast cancer. This work highlights the complementary role of HER2 and miR-21 biomarkers in breast cancer, highlighting their fundamental differences, but also their synergistic potential in clinical practice and translational research. A comparative analysis of detection methods highlights that, although conventional techniques remain the clinical standard, they are increasingly complemented by advanced technologies, such as digital PCR, optical and electrochemical biosensors. **Conclusions:** The emerging detection methods offer significant advantages in terms of sensitivity, rapidity and potential for miniaturization, but their clinical implementation is still limited by the lack of standardization and large-scale validation. A major bibliometric insight is the evident shift in publication output by country toward Asia, particularly China, India, and Iran, which currently show high publication output and extensive collaborative connectivity in the analyzed network. However, challenges remain in standardizing microRNA methods and the clinical validation of emerging technologies.

## 1. Introduction

Oncological biomarkers are a central element in modern oncology, contributing significantly to early diagnosis, prognosis assessment and the selection of personalized therapies. The development of molecular biology and genomic technologies has allowed the identification of tumor-specific molecular signatures, facilitating the transition to precision oncology [1,2,3,4,5]. In this context, biomarkers must meet strict criteria of sensitivity, specificity and reproducibility, while also having demonstrable clinical relevance [1,2,3].

In breast cancer, molecular biomarkers play a key role in the biological stratification of tumors and in guiding therapeutic decisions. Classical markers, such as estrogen receptors (ER) and progesterone receptors (PR), are widely used in the assessment of prognosis and response to hormonal therapy, while HER2 overexpression or amplification defines a distinct tumor subtype, characterized by increased aggressiveness, but also by sensitivity to targeted therapies [6,7,8]. Additionally, additional biomarkers, such as Ki-67 or multigenic tests, contribute to the assessment of recurrence risk and the personalization of adjuvant treatment [9,10,11].

Recent interest has extended to emerging biomarkers, particularly those detectable in biological fluids, such as circulating tumor DNA and microRNAs, which allow for minimally invasive and dynamic monitoring of disease [12,13]. Among them, miR-21 has emerged as one of the most studied onco-miRs, being involved in multiple oncogenic processes, including proliferation, invasion and treatment resistance [14,15,16].

Furthermore, the stability of miR-21 in circulation and its correlation with tumor burden and disease progression support its potential as a non-invasive biomarker for monitoring therapeutic response and early detection of recurrence [17,18].

The combined selection of HER2 and miR-21 biomarkers reflects an integrative approach to tumor biology. HER2 is a membrane receptor with tyrosine kinase activity, overexpressed in approximately 15–20% of breast cancers and associated with an aggressive phenotype, but treatable by targeted therapies [6,7,8]. In contrast, miR-21 is a post-transcriptional regulator of gene expression, detectable both intracellularly and in the circulation, providing information on tumor dynamics and the tumor microenvironment [14,15,16,17,18]. Hence, the combination of a tissue biomarker (HER2) and a circulating one (miR-21) allows for a more comprehensive characterization of the disease, integrating structural and functional information.

However, the clinical utility of these biomarkers directly depends on the accuracy of the detection methods. Conventional methods, such as immunohistochemistry for HER2 or PCR for microRNAs, have limitations related to preanalytical and interpretative variability [7,17,18]. On the other hand, advanced technologies, such as digital PCR or next-generation sequencing, offer increased sensitivity and allow the detection of biomarkers at very low concentrations [12,13].

The present work was designed as a hybrid systematic review with bibliometric mapping: (i) a PRISMA-guided systematic review to synthesize current detection strategies for HER2 and miR-21, and (ii) a bibliometric analysis using VOSviewer to reveal intellectual structure, trends and collaboration patterns. The bibliometric component was used as a complementary tool to contextualize the systematic synthesis, and critically analyze current methods for determining HER2 and miR-21 biomarkers, highlighting their advantages, limitations and clinical applicability, as well as future development directions in the field of precision oncology diagnostics.

## 2. Materials and Methods

### 2.1. Systematic Review

#### 2.1.1. Search Strategy

A comprehensive literature search was conducted in three major bibliographic databases: Web of Science Core Collection (WoSCC), PubMed, and Scopus.

The use of multiple databases was intended to maximize the identification of relevant studies, to broaden the literature retrieval and to reduce the risk of database-specific retrieval bias.

The initial search yielded 390 records in WoSCC, 98 records in PubMed, and 357 records in Scopus, after application of the respective search limits and filters. The retrieved records from the three databases were considered within the PRISMA-guided study-selection process, which resulted in a final corpus of 305 included publications. The search covered publications from 1 January 2016 to June 2026 and was restricted to full-text English-language documents (search date: 15 June 2026).

The following search query was employed for each database:

- WoSCC search string: TS = (“breast cancer”) AND TS = (“HER2” OR “ERBB2” OR “miR-21” OR “microRNA-21”) AND TS = (biosensor* OR aptasensor* OR immunosensor* OR “electrochemical biosensor*” OR “optical biosensor*” OR (“SPR” NEAR/3 biosensor*) OR (“surface plasmon resonance” NEAR/3 biosensor*) OR (“fluorescence” NEAR/3 detection) OR (“electrochemical” NEAR/3 detection)).

Document type: article, book chapter, proceeding paper, review. Retrieved records: 390.

- **PubMed** search string: (“breast cancer”[Title/Abstract] OR “breast neoplasms”[MeSH Terms]) AND (HER2[Title/Abstract] OR ERBB2[Title/Abstract] OR miR-21[Title/Abstract] OR microRNA-21[Title/Abstract]) AND (biosensor*[Title/Abstract] OR aptasensor*[Title/Abstract] OR immunosensor*[Title/Abstract] OR “electrochemical biosensor”[Title/Abstract] OR “optical biosensor”[Title/Abstract] OR electrochemical[Title/Abstract] OR SPR[Title/Abstract] OR “surface plasmon resonance”[Title/Abstract] OR fluorescence[Title/Abstract]).

Publication-type filters (human subjects only): Case Reports; Clinical Study; Clinical Trial; Clinical Trial Protocol; Conference Proceedings; Letter; Meta-Analysis; Multicenter Study; Observational Study; Review; Scoping Review; Systematic Review; Validation Study. Retrieved records: 98.

- **Scopus** search string: TITLE-ABS-KEY (“breast cancer”) AND TITLE-ABS-KEY (HER2 OR ERBB2 OR “miR-21” OR “microRNA-21”) AND TITLE-ABS-KEY (biosensor* OR aptasensor* OR immunosensor* OR “electrochemical biosensor” OR “optical biosensor” OR (SPR W/3 biosensor*) OR (“surface plasmon resonance” W/3 biosensor*) OR (fluorescence W/3 detection) OR (electro-chemical W/3 detection)).

Subject areas: Chemistry; Biochemistry, Genetics and Molecular Biology; Materials Science; Chemical Engineering; Medicine. Document type: article, book chapter, conference paper, review. Retrieved records: 357.

#### 2.1.2. Eligibility Criteria and Study Selection

**Figure 1:**
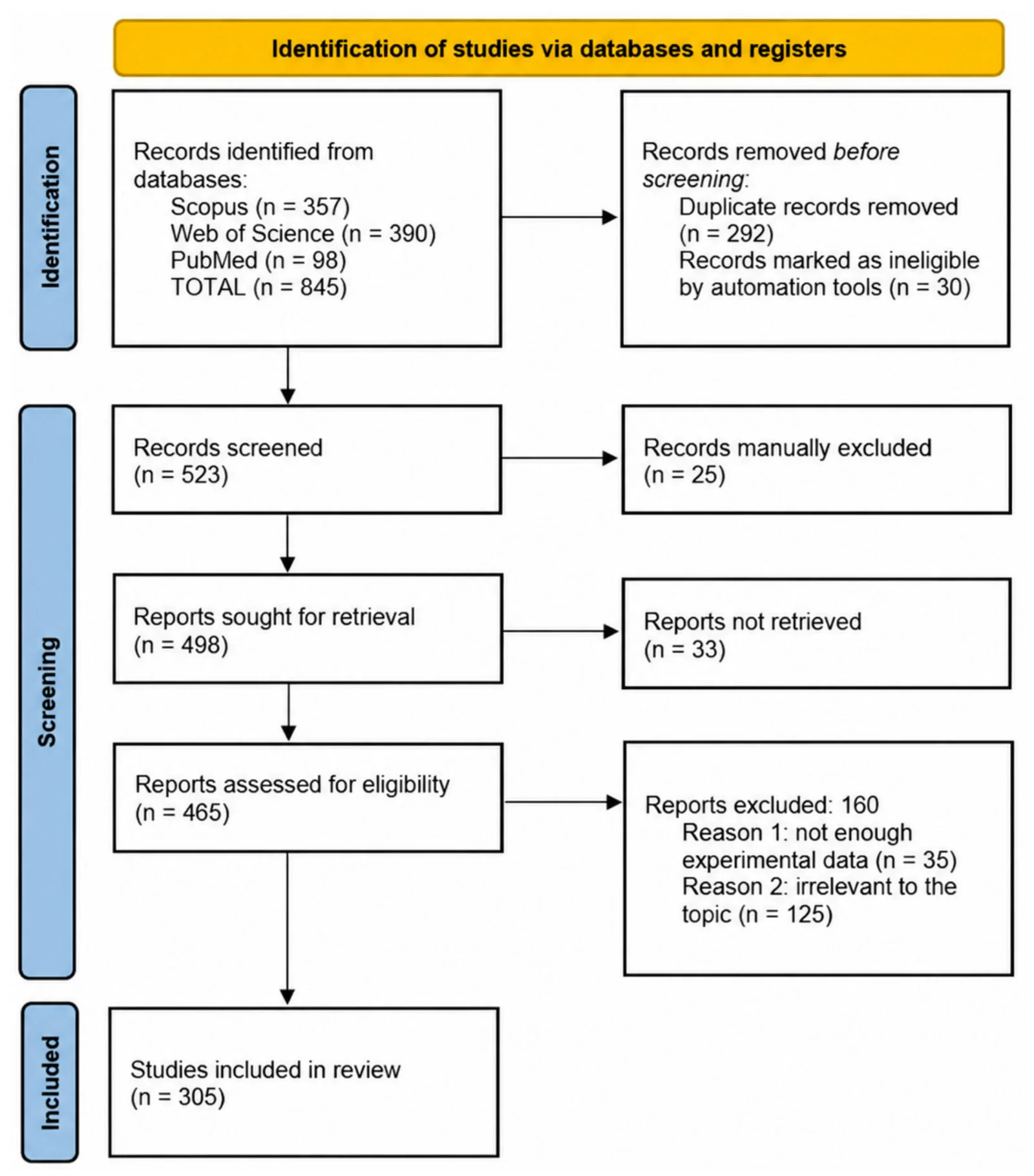
PRISMA flow diagram showing the visual chart of the identified documents, screened and included in this review.

Eligibility criteria were defined to identify publications relevant to the objectives of the hybrid review. Studies were considered eligible if they addressed breast cancer and investigated HER2/ERBB2 and/or miR-21 in the context of biomarker detection, analytical methods, or biosensor-based technologies. Eligible publications were required to provide sufficient information to characterize the biomarker, detection strategy, analytical platform, or clinical application. Original research articles were prioritized for the systematic evidence synthesis, whereas duplicate records and publications not directly relevant to the predefined review scope were excluded. All retrieved records underwent a sequential selection process involving duplicate removal, title and abstract screening, and full-text eligibility assessment. Records were excluded when they did not address the targeted biomarkers, breast cancer, or relevant detection strategies. The study-selection process was documented using the PRISMA flow diagram (Figure 1), resulting in a final corpus of 305 eligible publications.

This systematic review was carried out following the Preferred Reporting Items for Systematic Reviews (PRISMA) guidelines (Supplementary Material S1).

#### 2.1.3. Data Extraction

A structured data-extraction framework was applied to the 305 eligible publications to support the systematic evidence synthesis. Extracted variables included publication characteristics, target biomarker, breast cancer context or subtype when reported, detection method and biosensor platform, biological sample type, use of patient-derived samples, reported sample size, analytical performance characteristics, and evidence of clinical applicability. Additional information was recorded when available, including the use of independent comparator or reference methods, analytical validation parameters, and methodological characteristics such as blinding.

The extracted information provided the basis for the quantitative and descriptive characteristics reported in the subsequent sections, including the proportions of studies using patient-derived samples, sample-size distributions, and other methodological features. When a variable was not reported or could not be determined from the publication, it was recorded as not reported or unclear and was not inferred from the study context.

#### 2.1.4. Evidence Synthesis

Given the substantial heterogeneity of the included studies with respect to biosensor platforms, detection principles, biological matrices, study designs, and reported analytical outcomes, a formal quantitative meta-analysis was not considered appropriate. The extracted evidence was therefore synthesized descriptively and comparatively. Categorical study characteristics were summarized using frequencies and proportions, whereas analytical performance parameters were compared in the context of the respective biomarker, detection platform, biological matrix, and intended clinical application.

The systematic evidence synthesis focused on identifying major technological approaches, analytical strengths and limitations, and the degree of clinical and translational maturity of HER2 and miR-21 detection strategies.

### 2.2. Bibliometric Analysis: Data Analysis and Visualization

Bibliometric analyses were performed on the final corpus of 305 included publications using VOSviewer software (version 1.6.20) to investigate the intellectual structure, research trends, and collaboration patterns within the field of HER2 and miR-21 detection in breast cancer. To improve the consistency and accuracy of visualization mapping, a customized thesaurus file was developed and applied prior to the analysis. The thesaurus was used to merge synonymous terms, standardize different lexical variants (e.g., HER2, HER2/neu, ERBB2; miR-21, microRNA-21), and eliminate generic terms with limited bibliometric relevance. This procedure reduced redundancy and enhanced the visualization of thematic relationships within the dataset.

The retrieved records were analyzed using quantitative bibliometric techniques, including publication output analysis, keyword co-occurrence analysis, co-authorship analysis, and institutional and country collaboration mapping.

Keyword co-occurrence analysis was conducted to identify the main research themes and emerging topics within the scientific literature. The frequency of keyword occurrence and the strength of co-occurrence links were used to reveal conceptual relationships and thematic clusters.

Co-authorship analysis was performed at the author, organization, and country levels to evaluate collaboration patterns and research network structures. In the generated maps, node size represents the relative contribution of an entity, whereas link strength reflects the intensity of collaborative relationships between entities.

Network visualizations were generated using the association strength normalization method. The resulting maps enabled the identification of major research clusters, contributors with high publication output and network connectivity, and frequently occurring research themes associated with biosensor-based HER2 and miR-21 detection.

The interpretation of the bibliometric networks was based on node size, link strength, cluster composition, and spatial proximity between items, providing insights into the evolution and interdisciplinary nature of this research field.

The selected bibliometric indicators were chosen to provide complementary perspectives on the scientific landscape. The combined interpretation of these indicators allowed a comprehensive assessment of both the conceptual and social structures of research on HER2 and miR-21 biosensing technologies.

### 2.3. Structured Methodological and Translational Assessment

Given the heterogeneity of the included evidence, ranging from early-stage proof-of-concept biosensor studies to investigations involving clinical samples, a single risk-of-bias instrument was not considered applicable to the entire evidence base. Therefore, a structured methodological and translational assessment, specifically developed for this review, was applied across five predefined domains: (i) sample selection and clinical relevance, (ii) endogenous target relevance, (iii) analytical validation, (iv) use of an independent comparator or reference method, and (v) clinical applicability and discrimination.

Each domain was assessed using predefined criteria and categorized as low, moderate, high, or unclear concern. Insufficiently reported information was classified as unclear, while explicit methodological limitations were classified as great concern. Limitations related to the early developmental stage of biosensors were distinguished from methodological limitations. The framework provides a descriptive profile of methodological and translational characteristics and was not designed or validated as a formal risk-of-bias tool; no overall risk-of-bias score or composite judgment was derived.

Validated design-specific instruments, such as QUADAS-2, are appropriate for formal risk-of-bias assessment in eligible diagnostic-accuracy studies. However, the present review includes a heterogeneous evidence base comprising different study designs and early-stage biosensor development studies; therefore, the five-domain framework was used solely as a structured descriptive assessment across the full review corpus. This distinction is acknowledged as a methodological limitation of the present approach.

The resulting structured methodological concern profile of the 305 included studies is presented in Supplementary Material S2.

## 3. Results

### 3.1. Bibliometric Analysis

All bibliometric analyses presented in this section were performed on the 305 publications included in the final PRISMA corpus.

#### 3.1.1. Keyword Co-Occurrence Analysis

**Figure 2:**
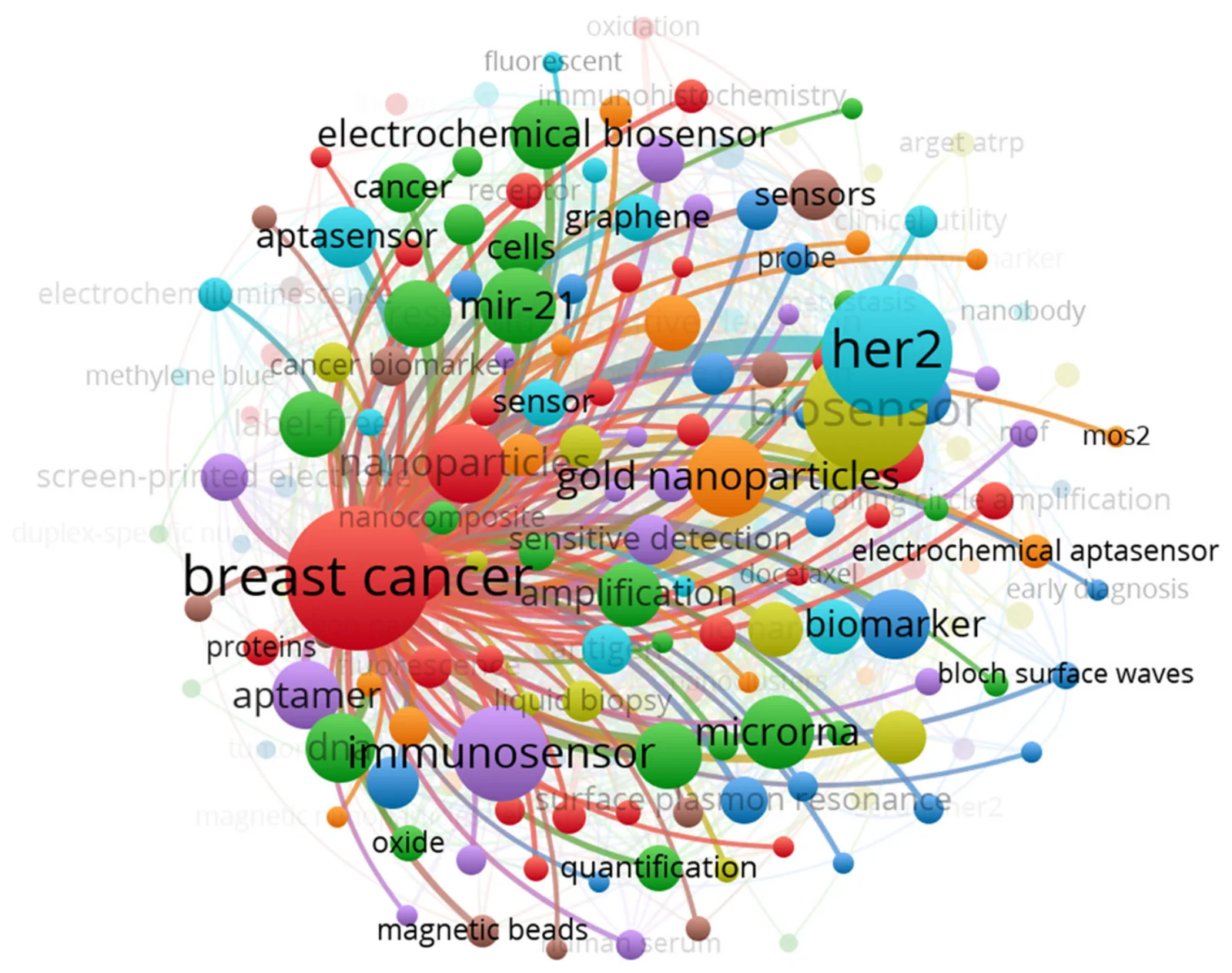
Co-occurrence network of author keywords in publications addressing HER2 and miR-21 detection in breast cancer.

Considering the publications selected through the PRISMA-guided process, keyword co-occurrence analysis was performed using VOSviewer to identify the main research themes and conceptual structure within the field of biosensor-based detection of breast cancer biomarkers (Figure 2). A minimum occurrence threshold of three was applied, resulting in the inclusion of 193 keywords out of 1463 total extracted terms, corresponding to approximately 13.2% of all keywords. The threshold was selected to reduce the influence of low-frequency terms while preserving the most representative concepts in the research field.

Prior to network construction, a manual keyword-cleaning procedure was performed to improve the reliability and interpretability of the analysis. This process included the standardization of synonymous terms, correction of spelling variants, merging of abbreviations and full-term expressions, and removal of non-informative or irrelevant keywords. This cleaning step substantially reduced network noise and improved thematic clarity.

The resulting keyword network reveals a highly interconnected conceptual structure dominated by several core themes. The largest and most central node is breast cancer, confirming that the overall literature is strongly focused on diagnostic and analytical strategies for breast cancer detection. This term acts as the principal hub connecting multiple thematic subdomains.

A second major thematic axis is centered around HER2, which emerges as one of the most frequently investigated biomarkers by publication count in the network. Its large node size and extensive connectivity indicate that HER2-targeted biosensing represents one of the most intensively investigated research directions in the field. Strong associations between HER2 and terms such as biosensor, electrochemical aptasensor, gold nanoparticles, and surface plasmon resonance suggest a major focus on sensitive analytical platforms for HER2 detection.

Another dominant cluster is centered around microRNA and particularly miR-21, reflecting the increasing importance of nucleic acid biomarkers in breast cancer diagnostics. The strong connectivity between these terms and nodes such as amplification, probe, and quantification highlights the growing use of amplification-assisted sensing strategies for ultrasensitive detection of circulating miRNA biomarkers.

The network also highlights a strong technological emphasis on biosensor engineering. Key nodes including biosensors, electrochemical biosensors, immunosensors, aptasensors, and sensors indicate that electrochemical and affinity-based sensing technologies dominate the field. These technologies appear to serve as the principal analytical platforms for biomarker detection. Nanotechnology-related terms also occupy central positions in the network. Keywords such as gold nanoparticles, graphene, nanoparticles, nanocomposite, magnetic beads, and nanosheets form a highly interconnected technological cluster. Their prominence reflects the critical role of nanomaterials in enhancing signal amplification, sensitivity, and selectivity in biosensing systems.

In addition, several clinically relevant emerging themes are visible, including liquid biopsy, tumor marker, cancer diagnosis, and nanobody, indicating increasing research interest in minimally invasive diagnostic strategies and next-generation recognition elements.

#### 3.1.2. Co-Authorship Network Analysis

**Figure 3:**
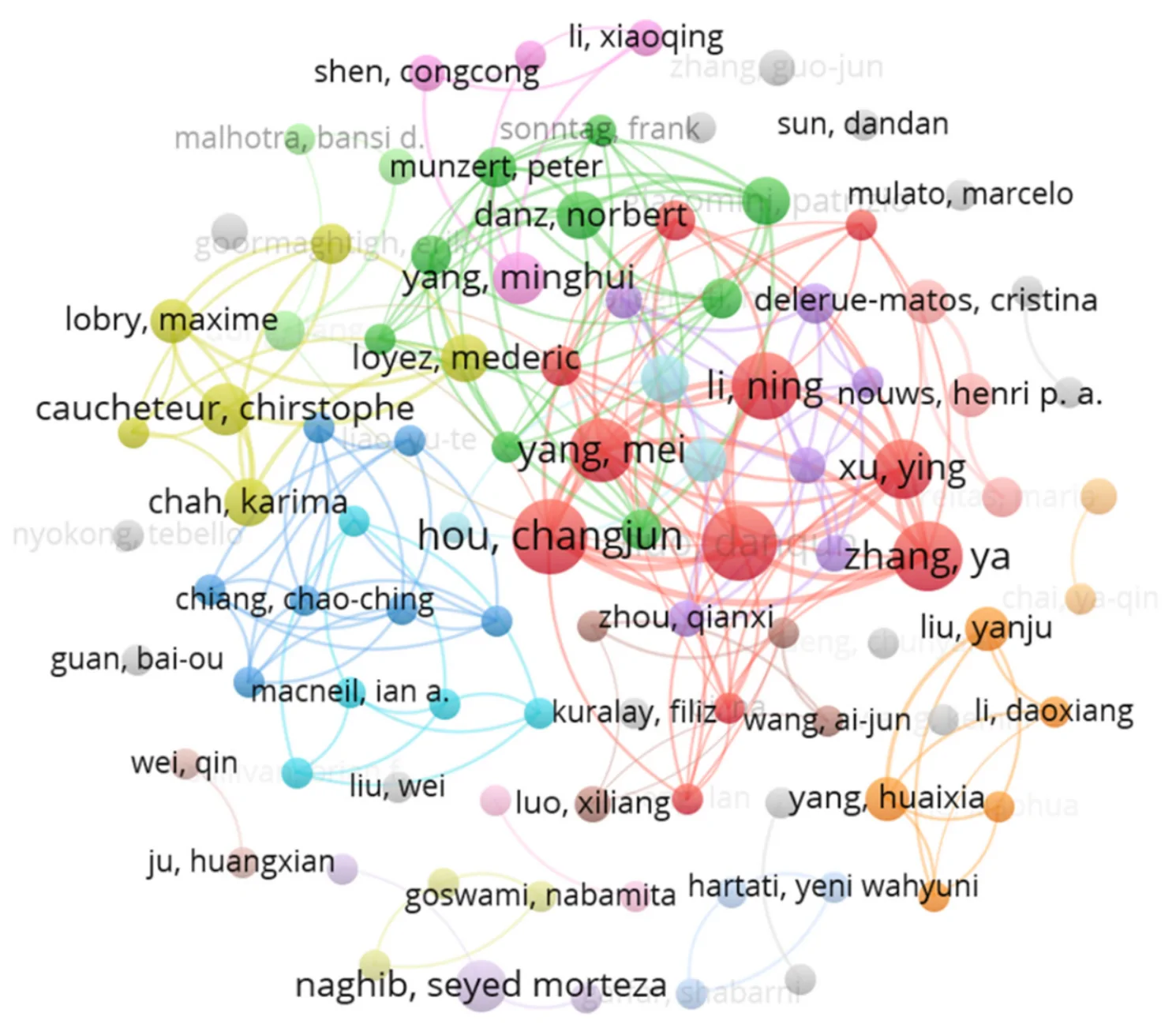
Synthesis of the author co-authorship network of publications related to HER2 and miR-21 detection in breast cancer.

A co-authorship analysis was performed using VOSviewer to explore collaboration patterns among researchers working on HER2 and miR-21 detection in breast cancer. A minimum threshold of three publications per author was applied, while no restriction was imposed on the number of citations. Out of 1903 identified authors, 96 met the inclusion criteria and were retained for analysis, thereby improving the interpretability of the network structure. As presented in Figure 3, the co-authorship network analysis revealed a highly interconnected cluster-structured authorship landscape in the field of biosensor-based detection of breast cancer biomarkers, particularly HER2/ERBB2 and miR-21. The co-authorship network revealed several research groups characterized by high publication output and large co-authorship connectivity. Hou Changjun, Zhang Ya, Li Ning, Yang Mei and Xu Ying emerge as the authors associated with large node sizes and extensive co-authorship connectivity in the network, indicating their central roles in scientific productivity and collaborative influence. These authors occupy highly connected positions in the co-authorship network, with strong links to multiple co-authors. Their network positions are associated with extensive co-authorship across studies addressing biosensor development.

In particular, Hou Changjun appears as one of the most productive authors, characterized by a large node size and extensive collaborative links across multiple clusters, indicating strong interdisciplinary collaboration and extensive connectivity across multiple research clusters.

These clusters may reflect geographically concentrated teams or research communities focused on specific biosensing technologies, including optical biosensors, fiber-optic sensing, and advanced nanomaterial-based platforms.

The network also reveals the presence of several smaller peripheral clusters and isolated authors. These nodes likely represent emerging contributors or specialized researchers with more focused collaboration patterns. Despite their smaller influence and more limited connectivity, such authors contribute to the diversification and expansion of research themes within the field.

#### 3.1.3. Organizational Collaboration Network Analysis

**Figure 4:**
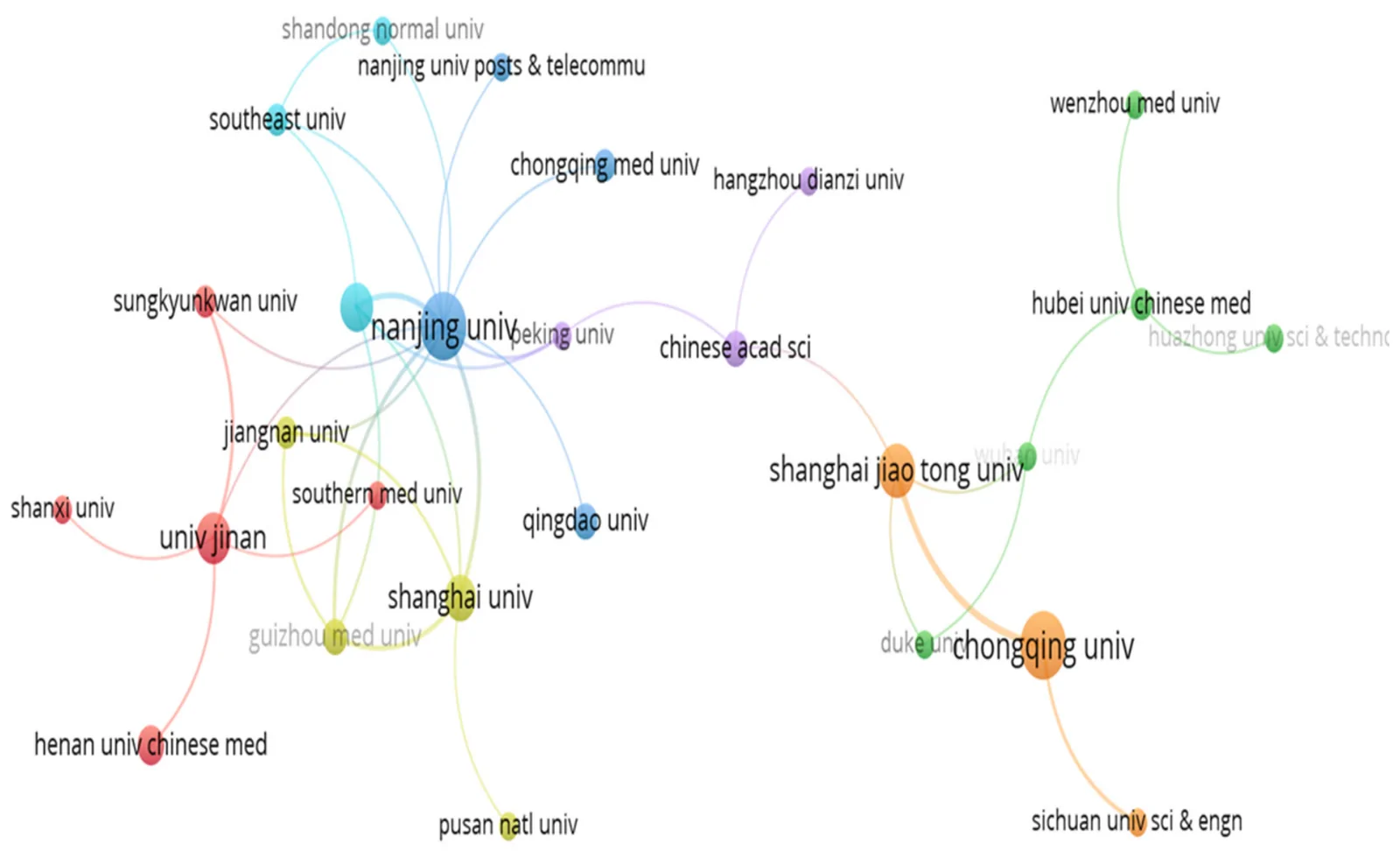
Synthesis of institutional network of publications related to HER2 and miR-21 detection in breast cancer.

To investigate institutional collaboration patterns in research related to HER2 and miR-21 detection in breast cancer, a co-authorship analysis at the organizational level was performed. A minimum threshold of three publications per organization was applied, while no minimum citation requirement was imposed. Out of 534 identified organizations, 72 met the selection criteria and were included in the collaboration network analysis. The selected threshold enabled the identification of the most active institutions while reducing the influence of organizations represented by a single publication. This approach facilitated a clearer visualization of collaborative relationships and institutional research networks within the field (Figure 4).

The organizational collaboration network provides a detailed overview of institutional interactions within the research field of biosensor-based detection of breast cancer biomarkers, particularly HER2/ERBB2 and miR-21. Compared with the country collaboration network, the institutional network appears more fragmented and cluster-oriented, reflecting localized collaboration patterns among universities and research centers. Nevertheless, several institutions showing large node sizes and strong collaborative connectivity emerge as central nodes in the network, particularly from China.

Chinese academic institutions account for a large share of the observed institutional publication output and collaborative links in the network. The overall structure suggests that research productivity and collaboration are concentrated within a relatively limited number of highly active universities, with several institutions functioning as central nodes that connect multiple collaboration clusters.

Nanjing University is characterized by high connectivity, a large node size, and a central position within the institutional collaboration network (blue cluster). Its strong collaborative links with institutions such as Southeast University, Chongqing Medical University, Qingdao University, Beijing University, and Nanjing University of Posts and Telecommunications indicate extensive participation in multidisciplinary research networks. The centrality of Nanjing University suggests a significant contribution to biosensor development, nanotechnology-driven sensing platforms, and advanced analytical diagnostics.

Another strong institutional hub is Chongqing University, which appears as a separate but highly active collaboration cluster (orange cluster), demonstrating particularly strong collaborative ties with Shanghai Jiao Tong University, forming one of the strongest institutional partnerships observed in the network. This pattern reflects strong co-authorship connectivity within biosensor engineering, electrochemical sensing, and nanomaterial-based analytical platforms.

Shanghai Jiao Tong University also occupies a central position in the institutional collaboration network. Its position as a connector between multiple institutions, including Wuhan University and Duke University, reflects extensive connectivity within the institutional collaboration network.

The network also identifies the University of Jinan as a well-connected institutional node, with notable collaborative links to Jiangnan University, Southern Medical University, Henan University of Chinese Medicine, and Shanxi University. Its central position suggests active contributions in biosensor fabrication, analytical chemistry, and biomedical sensing applications. This cluster appears to be characterized by strong domestic collaboration within China, emphasizing regional scientific integration.

The presence of non-Chinese institutions such as Duke University and Pusan National University indicates that international institutional collaboration is present but remains comparatively limited within the current network. This suggests that, despite the global nature of biosensor research, institutional collaborations in this specific domain remain predominantly concentrated within national or regional scientific ecosystems, particularly in China.

#### 3.1.4. Country Collaboration Network Analysis

To evaluate the geographical distribution of research activities and international collaboration patterns, a co-authorship analysis at the country level was conducted. A minimum threshold of one publication per country was applied, while no restriction was imposed on citation counts. Consequently, all 50 countries identified within the dataset met the selection criteria and were included in the analysis.

The inclusion of all contributing countries enabled a comprehensive assessment of the global research landscape and facilitated the identification of international collaboration networks associated with HER2 and miR-21 detection in breast cancer.

**Figure 5:**
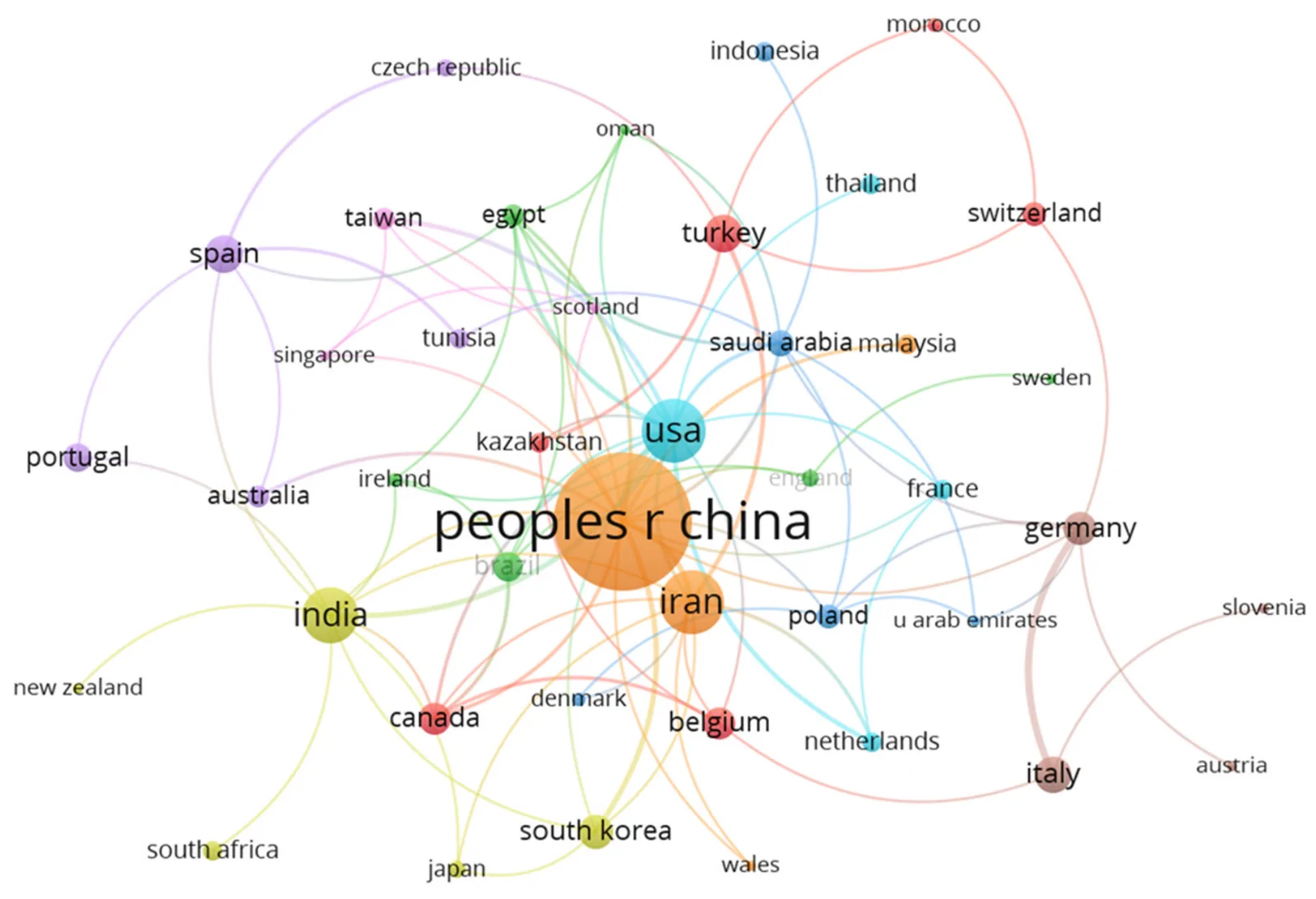
Synthesis of international co-authorship network of countries contributing to research on HER2 and miR-21 detection in breast cancer.

As presented in Figure 5, the country collaboration network revealed a highly interconnected and globally distributed research ecosystem in the field of biosensor-based detection of breast cancer biomarkers, with a particular focus on HER2/ERBB2 and miR-21.

Among all contributing countries, the People’s Republic of China exhibits both the largest node size and the highest number of collaborative links, indicating extensive international connectivity within the network. This finding strongly suggests the rapid expansion of research focused on high-sensitivity biosensing platforms for early cancer detection, nanotechnology and point-of-care diagnostic systems.

The United States represents the second major hub and occupies a highly connected bridging position within the collaboration network. Strong collaborative relationships with China, Saudi Arabia, France, Turkey, and the Netherlands highlight its role as an integrative force in the global research landscape. In contrast to China’s volume-driven dominance, the United States appears to contribute predominantly through extensive interdisciplinary collaborations, particularly at the interface of biosensor engineering, translational diagnostics, and clinical validation. This pattern reflects the strong involvement of U.S. institutions in bridging fundamental biosensor innovation with practical biomedical applications.

A particularly noteworthy finding is the strong representation of emerging scientific contributors from Asia and the Middle East, especially Iran and India, both of which demonstrate substantial research productivity and high network connectivity. Their increasing visibility reflects significant advancements in cost-effective biosensor development, microfluidic technologies, and scalable diagnostic platforms.

The European research landscape appears more distributed and less centralized, consisting of multiple interconnected collaboration clusters rather than a single dominant hub. Within Europe, Germany, Italy, France, Belgium, and the Netherlands show comparatively high publication output and network connectivity.

Notably, Germany and Italy demonstrate one of the strongest bilateral collaboration links in the network, indicative of long-standing partnerships in analytical chemistry, biomedical engineering, and translational diagnostic research. France and the Netherlands further strengthen the European contribution by serving as key connectors between European, North American, and Asian research communities, thereby facilitating international knowledge exchange. Turkey occupies an intermediary position within the collaboration network, facilitating collaboration across geographically distinct research communities.

Several countries, including Portugal, Morocco, Slovenia, Austria, New Zealand, South Africa, Indonesia, and the Czech Republic, are positioned at the periphery of the network. Although these countries contribute less extensively in terms of publication output and collaborative intensity, their presence reflects the expanding global dissemination of biosensor research and the growing international relevance of biomarker-based breast cancer diagnostics.

It should be noted that bibliometric indicators used herein—publication count, citation count, and collaboration centrality—should be interpreted strictly in terms of publication output, citation visibility, and collaboration patterns rather than scientific quality, technological innovation, clinical validity, or translational impact. High publication output may also be influenced by national funding priorities, institutional size, and publication practices.

#### 3.1.5. The Most Cited Papers Related to the Topic

Table 1 presents the top 10 most cited publications within the dataset, providing an overview of the most highly cited publications in the field. These papers accumulated 1551 citations in total, with an average of 155.1 citations per article.

The most cited document in the dataset was the original article titled “Bimetallic Single-Atom Nanozyme-Based Electrochemical-Photothermal Dual-Function Portable Immunoassay with Smartphone Imaging” authored by Yunsen Wang et al. in 2024 [19], describing the development of a portable device based on an electrochemical-photothermal bifunctional immunoassay detection platform designed to achieve reliable detection of HER2. The second most-cited publication was the review paper authored by M. Zubair in 2021 [20], which offered consolidated information on currently available diagnosis and treatment options in breast cancer, describing advanced biomarkers for the development of state-of-the-art early screening and diagnostic technologies. The third position was occupied by a research article describing the design of a label-free and simple electrochemical sensing platform to detect microRNA-21 (miR-21) with high sensitivity by using MoS2 nanosheets functionalized with thionine and gold nanoparticles (MoS2-Thi-AuNPs), published by Dan Zhu et al. in 2017 [21].

## 4. Discussion

### 4.1. Bibliometric Analysis: Data Analysis and Visualization

The main research themes and conceptual structure within the field of biosensor-based detection of breast cancer biomarkers were identified through the visualization maps. In terms of keyword co-occurrence analysis, it was demonstrated that research in this field is primarily organized around three major conceptual pillars: clinically relevant biomarkers (HER2 and miR-21), biosensing technologies (electrochemical and affinity-based platforms), and nanomaterial-assisted signal amplification strategies. The high density of interconnections among these themes reflects the strongly multidisciplinary nature of this research area, integrating oncology, nanotechnology, analytical chemistry, and biosensor engineering to advance precision diagnostics in breast cancer. Early research primarily focused on nanomaterial-assisted sensor development, with gold nanoparticles, ionic liquids, metal–organic frameworks, and modified electrodes emerging as dominant technological platforms [21,26]. Starting from 2018, research attention progressively shifted toward clinically relevant biomarkers, particularly the HER2 antigen and microRNAs, highlighting the increasing importance of molecular diagnostics in breast cancer detection [27,28]. From 2019 onward, electrochemical aptasensors became a central research direction due to their high sensitivity, selectivity, and suitability for point-of-care applications. These findings indicate a transition from material-driven sensor optimization toward clinically oriented biosensing strategies for early diagnosis and biomarker detection in breast cancer [20].

Overall, the co-authorship network highlights a collaborative and multidisciplinary scientific landscape characterized by highly productive authors and well-established research groups. The strong clustering pattern suggests that research in biosensor-based breast cancer diagnostics is organized around specialized collaborative communities with distinct scientific expertise. At the same time, the presence of multiple intercluster connections indicates increasing integration across research groups, reflecting the multidisciplinary nature of research in biosensor technologies. These findings underscore the importance of scientific collaboration in accelerating the development of highly sensitive, selective, and clinically applicable biosensing platforms for precision oncology. Chinese universities show high publication output and extensive collaborative connectivity within the network. Cluster analysis further reveals the presence of distinct collaboration communities with both geographical and thematic characteristics. While the USA is the second major cluster highlighting multidisciplinary and translational research, the European clusters appear more distributed and less centralized, primarily linked to analytical and clinical applications.

The bibliometric patterns observed are unlikely to be random and may reflect broader drivers related to technological development, complementary scientific expertise, research priorities, and evolving clinical needs. The emergence of collaboration clusters may be associated with the multidisciplinary nature of the field, bringing together complementary expertise in areas such as analytical chemistry, nanomaterials, molecular biology, and clinical oncology, as well as with institutional and geographic proximity. Inter-cluster collaborations may further reflect the increasing need for broader interdisciplinary and international cooperation to support technological development and translational validation. However, bibliometric indicators reflect productivity and connectivity, not necessarily scientific quality.

Our findings suggest three actionable directions: (1) Bridging clusters: Future progress requires intentional bridging between materials science and clinical oncology, as the current network shows low inter-cluster density. Consortia involving analytical chemists, oncologists, and regulatory scientists are needed. (2) From single to multi-marker panels: Bibliometric data during 2023–2025 align with clinical evidence that no single marker reaches sufficient AUC (>0.90), requiring cross-technology collaboration. (3) Standardization before innovation: the field needs large multicenter validation (*n* > 300), certified reference materials, and adherence to MIQE and REMARK guidelines.

### 4.2. Biological Characteristics and Underlying Molecular Mechanisms

#### 4.2.1. Molecular Heterogeneity of miR-21 in Genetically Defined Breast Cancer Subgroups

HER2 (Human Epidermal Growth Factor Receptor 2), also known as ERBB2, is a transmembrane receptor with tyrosine kinase activity, involved in the regulation of cell proliferation and survival. Its activation determines the initiation of intracellular signaling cascades, such as the PI3K/AKT and MAPK pathways, essential for tumor [29,30,31,32]. HER2 overexpression or amplification, present in approximately 15–20% of breast cancer cases, is associated with an aggressive phenotype and an increased risk of recurrence and metastasis [29,30,31,32,33]. HER2 overexpression or amplification, present in approximately 15–20% of breast cancer cases, is associated with an aggressive phenotype and an increased risk of recurrence and metastasis [30,31,32].

In contrast, miR-21 is a non-coding microRNA involved in the post-transcriptional regulation of gene expression. It acts by inhibiting tumor suppressor genes, such as PTEN, PDCD4 or SPRY2, facilitating the activation of oncogenic pathways and promoting proliferation, invasion and resistance to apoptosis [34,35,36]. Unlike HER2, which is a surface protein marker, miR-21 acts at a fine molecular level, having the ability to simultaneously regulate multiple cellular pathways [14,33,34,35,36,37,38,39]. Unlike HER2, which is a surface protein marker, miR-21 acts at a fine molecular level, having the ability to simultaneously regulate multiple cellular pathways [14,35,36,37,38].

A notable difference between the two biomarkers lies in the level at which they intervene in tumor biology: HER2 is a direct determinant of oncogenic signaling at the membrane level, while miR-21 functions as a global regulator of gene expression, indirectly influencing numerous cellular processes. This complementarity suggests that their combined use may provide a more complete picture of tumor behavior.

However, molecular heterogeneity is evident, and the diagnostic and prognostic significance of miR-21 is subtype-dependent. The diagnostic and prognostic relevance of miR-21 varies according to the molecular context of breast cancer. Available clinical and molecular data indicate that miR-21 expression is associated with aggressive disease characteristics and that its prognostic significance is particularly evident in aggressive subgroups, including triple-negative breast cancer, while analyses of TCGA and METABRIC have also demonstrated associations between miR-21 expression and HER2-positive/HER2-enriched tumors [40]. Importantly, most biosensor studies included in our review did not stratify miR-21 performance according to molecular subtype or TP53 status, representing an important translational gap and a potential source of spectrum bias. We therefore emphasize that miR-21 should not be interpreted as a uniform biomarker across breast cancer, and that future biosensor studies should incorporate molecular stratification and, where appropriate, multiplexed detection strategies.

#### 4.2.2. Clinical Relevance and Prognostic/Predictive Value

Clinically, HER2 is one of the best-validated biomarkers in oncology, with both prognostic and predictive value. HER2-positive tumors are characterized by increased aggressiveness, but also by a favorable response to targeted therapies, such as trastuzumab or other monoclonal antibodies and antibody–drug conjugates [7,8,13,41]. Determining HER2 status is mandatory in clinical practice, being essential for choosing treatment [7,42,43].

Instead, miR-21 is considered an emerging biomarker, with predominantly prognostic value. Elevated levels of miR-21 are correlated with advanced disease stages, metastasis, treatment resistance, and reduced survival [14,15,16,17,18,40,44,45]. Although not yet routinely used in clinical guidelines, miR-21 shows significant potential as a non-invasive biomarker, particularly for dynamic monitoring of disease through measurements in plasma or serum [16,17,18,46].

In comparison, HER2 has a clear role in guiding therapy (predictive biomarker), while miR-21 provides additional information about disease progression and response to treatment (prognostic and monitoring biomarker). The integration of these two markers may improve patient stratification and therapy personalization.

**Figure 6:**
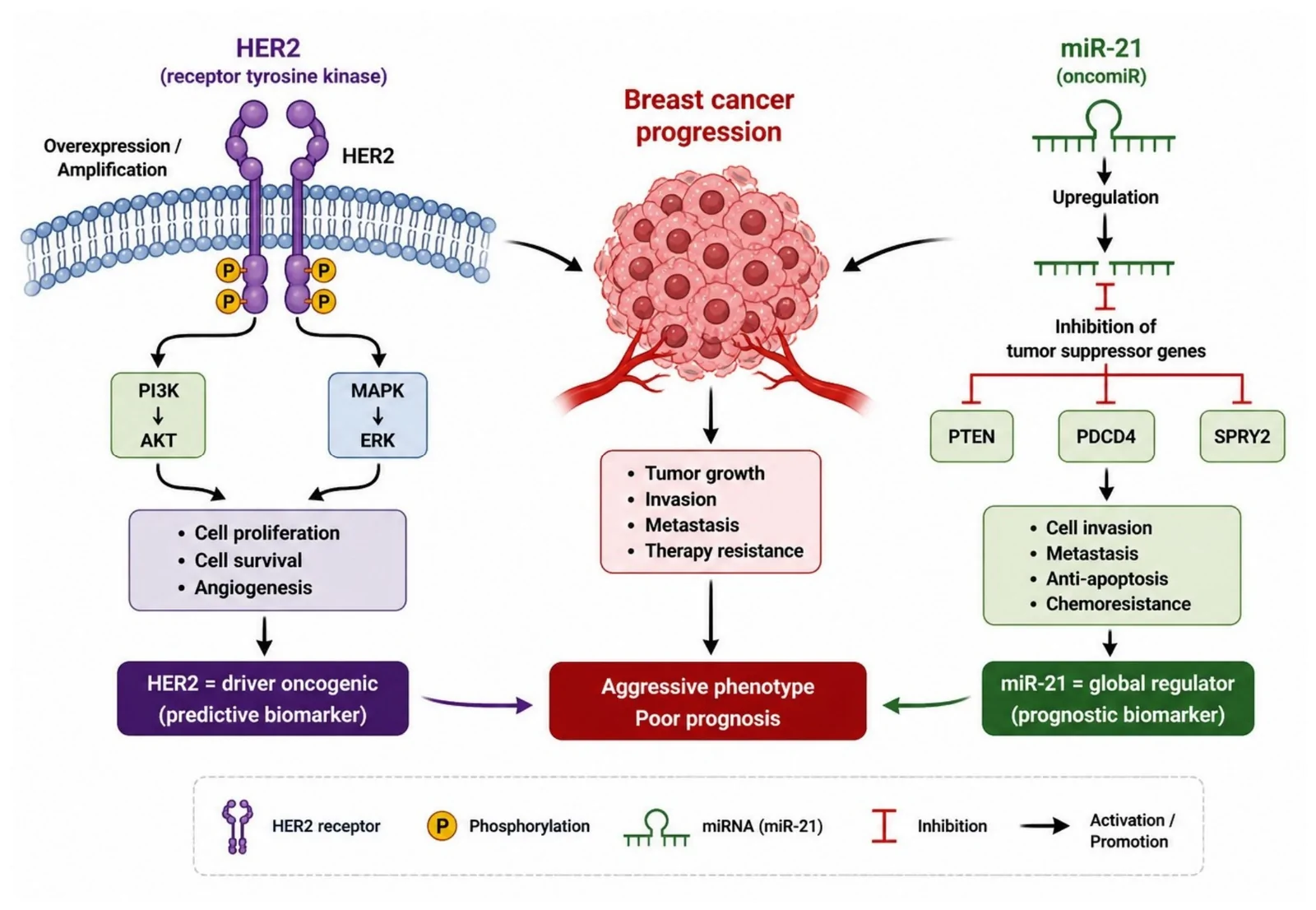
Molecular roles of HER2 and miR-21 in breast cancer progression.

The interaction between HER2-mediated signaling pathways and miR-21-associated post-transcriptional regulatory mechanisms is schematically illustrated in Figure 6, highlighting their complementarity in breast cancer progression.

HER2 activation promotes oncogenic signaling through PI3K/AKT and MAPK pathways, leading to increased cell proliferation, survival, and angiogenesis. In contrast, miR-21 acts as an onco-miR by inhibiting tumor suppressor genes such as PTEN, PDCD4, and SPRY2, thereby enhancing invasion, metastasis, and therapy resistance. The combined effects contribute to tumor progression and poor prognosis [29,30,31,32,34,35,36,37,41,42,44,47,48].

#### 4.2.3. Sample Types and Biomarker Dynamics

Another major aspect that differentiates HER2 from miR-21 is the type of sample required for determination. HER2 is mainly assessed in tumor tissue by techniques such as immunohistochemistry or in situ hybridization, providing a static image of protein expression at the time of biopsy [7,42,43,49,50].

In contrast to this, miR-21 can be detected both in tissues and in biological fluids (plasma, serum), in free form or encapsulated in exosomes, which allows for non-invasive and repeated assessment over time [43,46,51]. This feature gives miR-21 a major advantage in monitoring disease progression and response to treatment.

Therefore, HER2 provides structural and tissue-specific information, while miR-21 reflects systemic tumor dynamics. This difference highlights the complementary potential of the two biomarkers in precision oncology.

#### 4.2.4. Advantages, Limitations and Complementarity

Direct comparison of the two biomarkers highlights fundamental differences, but also an important synergistic potential.

HER2 benefits from clinical standardization, validated methods and direct therapeutic applicability, but is limited by the invasive nature of the determination and the impossibility of frequent dynamic monitoring. In addition, tumor heterogeneity can influence the accuracy of the assessment [7,43,49,50,51,52].

On the other hand, miR-21 offers the advantage of non-invasive detection and high sensitivity in reflecting disease progression, but is limited by the lack of standardization of determination methods and biological variability [16,17,18,46,53,54,55,56].

Thus, the combined use of HER2 and miR-21 can overcome the individual limitations of each biomarker: HER2 provides essential information for therapy selection, and miR-21 allows for real-time monitoring of therapeutic response and tumor progression. This integrative approach is consistent with the principles of precision oncology and represents a promising direction for future research.

To better understand the fundamental differences between the HER2 and miR-21 biomarkers, the main biological, clinical, and analytical characteristics are summarized in Table 2.

As can be seen, HER2 is a protein biomarker with a direct role in oncogenic signaling and a well-established clinical applicability, while miR-21 acts as a post-transcriptional regulator, providing additional information on tumor dynamics. The differences between the two biomarkers highlight their complementary nature, suggesting that their combined use may significantly improve the accuracy of diagnosis and disease monitoring [13,29,30,31,32,33,34,35,36,37,38,39,40,41,42,43,46,47,48,49,50,51,52,53,54,55,56,57,80,81].

### 4.3. Detection Strategies for miR-21 and HER2 Biomarkers—A Comparative Analysis

#### 4.3.1. Conventional Methods: State of Current Clinical Practice

The determination of HER2 and miR-21 biomarkers relies on a diverse range of analytical methods, each with distinct characteristics in terms of sensitivity, specificity and clinical applicability. Conventional methods remain the current standard, especially for HER2, due to extensive clinical validation and integration into international guidelines [7,42,43,54,55,56].

Immunohistochemistry (IHC) is the first-line method for assessing HER2 protein expression, being widely used in the diagnosis of breast cancer [7,42,43,51,52]. This technique offers the advantage of correlating biomarker expression with tissue architecture, but has limitations related to inter-observer variability and the influence of preanalytical factors [49,50,51,52]. In this context, standardization of procedures and use of ASCO/CAP guidelines are essential to ensure reproducibility.

Fluorescence in situ hybridization (FISH) is considered the reference method for determining ERBB2 gene amplification, having superior specificity compared to IHC [7,42,43,51,52]. However, high costs and the need for specialized equipment limit its use in certain clinical contexts.

Regarding miR-21, amplification-based techniques, such as qRT-PCR and digital PCR, are the most widely used [54,55,56]. qRT-PCR offers high sensitivity and is relatively affordable, but quantification is dependent on normalization and sample quality. Digital PCR, on the other hand, allows absolute quantification and detection of subtle variations and is considered a reference method for circulating biomarkers [56].

Comparatively, conventional methods for HER2 are better standardized and clinically implemented, while for miR-21 they are still in the process of extensive clinical validation.

The methods used for determining the HER2 and miR-21 biomarkers, along with their analytical sensitivity, specificity, reproducibility, limit of detection, clinical validation, cost, turnaround time, current regulatory status, and barriers to routine clinical implementation are summarized in Table 3.

Comparative analysis highlights that conventional methods, such as immunohistochemistry and in situ hybridization, remain the clinical standard due to extensive validation and high specificity. However, emerging methods, including optical and electrochemical biosensors, offer significant advantages in terms of sensitivity and feasibility of implementation in portable systems, suggesting significant potential for future applications [58,59,60,61,62].

As shown, amplification-based methods, such as qRT-PCR and digital PCR, offer the highest sensitivity and are widely used in research and some clinical applications. At the same time, emerging technologies, such as biosensors, have the advantage of rapid detection and the possibility of miniaturization, but require further validation for clinical implementation [69,70,71,72].

#### 4.3.2. Advanced Optical Methods

Modern optical methods extend the detection capacity of biomarkers by increasing sensitivity and the possibility of multiplex analysis.

ELISA tests and spectrophotometric techniques are used to quantify the soluble form of HER2 (sHER2) in serum or plasma. They offer the advantage of minimally invasive analysis and low cost, but have lower sensitivity compared to tissue methods and do not provide information on gene status or cellular localization [11,82,83,84].

FISH techniques applied to microRNAs (miRNA-FISH) allow visualization of miR-21 expression in a tissue context, providing information on cellular distribution and tumor heterogeneity. However, the method is complex, expensive and does not allow absolute quantification [69,70,71].

Optical biosensors, including those based on Surface Plasmon Resonance (SPR) or localized surface plasmon resonance (LSPR), represent an emerging direction in HER2 and miR-21 detection. These technologies allow for real-time, label-free detection with very high sensitivity [57,58,77]. Also, methods based on photoluminescence and quantum dots offer the possibility of multiplexing and detection at very low concentrations [59,60].

Compared to conventional methods, advanced optical techniques offer superior analytical performance, but are limited by cost, complexity, and lack of extensive clinical validation.

**Figure 7:**
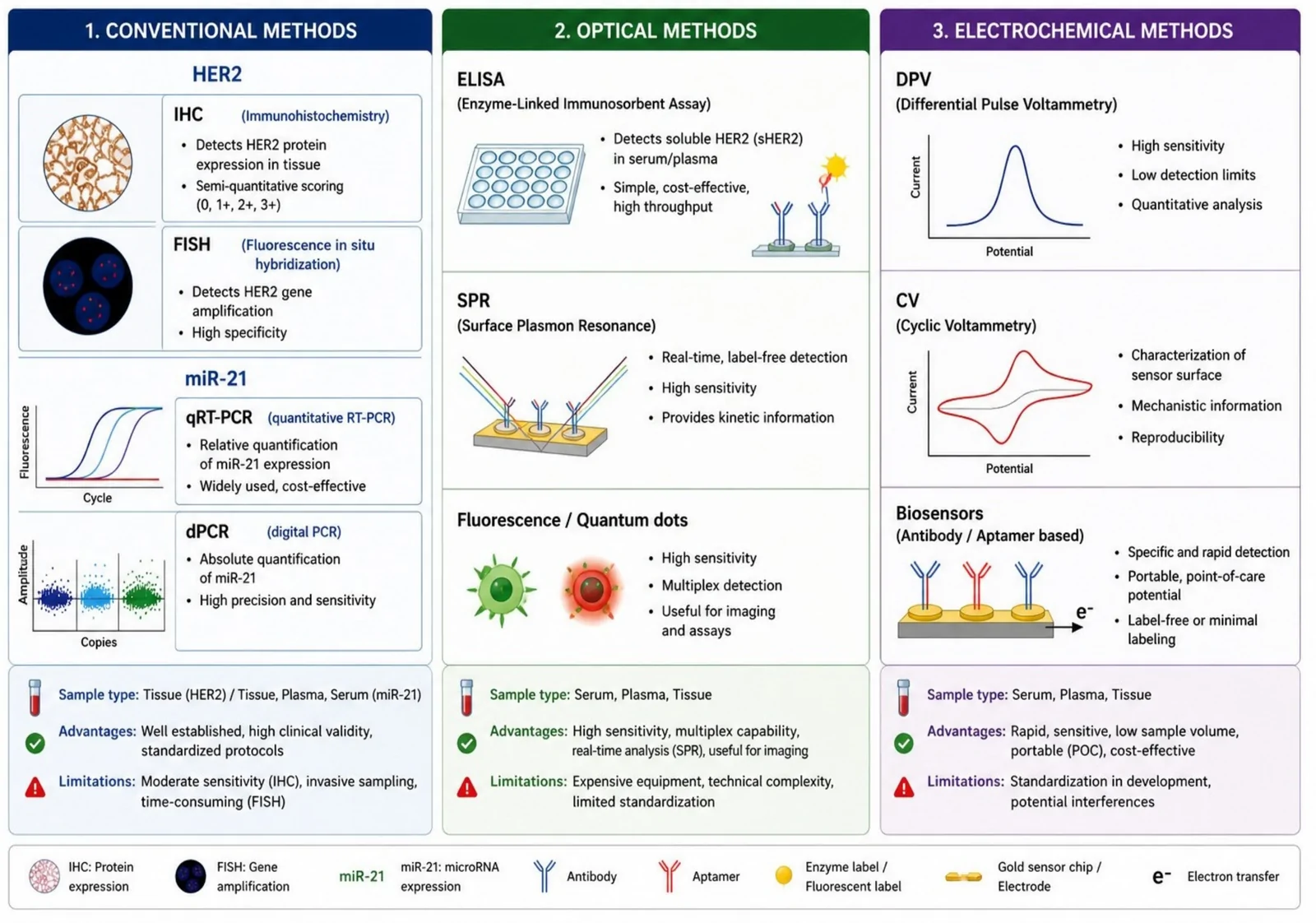
Overview of the main detection methods for HER2 and miR-21 biomarkers.

The main detection methods for HER2 and miR-21 biomarkers, including conventional, optical, and electrochemical techniques, are summarized in Figure 7.

Conventional methods such as immunohistochemistry (IHC) and fluorescence in situ hybridization (FISH) are standard for HER2 evaluation, while qRT-PCR and digital PCR are widely used for miR-21 detection. Emerging optical and electrochemical biosensors provide enhanced sensitivity and potential for rapid, point-of-care applications [58,59,60,61,62,63,64,65,66,67,69,70,71,72,73,74,75,82,83,84,85].

#### 4.3.3. Electrochemical Methods and Biosensors

Electrochemical methods represent one of the most promising directions for the development of rapid, sensitive and portable detection systems.

Differential pulse voltammetry (DPV) offers high sensitivity, with detection limits in the pico- or femtomolar range, being used in particular in biosensors for HER2 [19,24,25,26,27,28,68,72,75]. DPV allows for rapid analysis and low costs, but clinical applicability is still limited by the lack of standardization and biological matrix interferences [68,72,75].

Cyclic voltammetry (CV) is mainly used for the characterization and optimization of biosensors, providing information about electrochemical mechanisms and surface processes [61,63,75,78]. Although CV is less sensitive for quantification, it is essential for the development and optimization of analytical platforms.

Electrochemical biosensors functionalized with antibodies or aptamers combine biological specificity with electrochemical sensitivity. Antibodies offer high affinity but can be limited by stability and cost, while aptamers offer advantages such as increased stability, reproducibility, and low cost [63,64,65,66].

The integration of nanomaterials (gold nanoparticles, graphene) significantly increases the performance of these systems [21,24,26,28,62].

#### 4.3.4. Overall Comparative Analysis of the Methods

Direct comparison of detection methods reveals major differences depending on the biomarker and clinical or research purpose.

For HER2, tissue methods (IHC and FISH) remain the clinical standard due to their extensive validation and their direct role in guiding therapy. However, they are limited to point-of-care and invasive assessments. Serum methods (ELISA) and biosensors offer the possibility of dynamic monitoring, but require further validation before clinical implementation [7,42,43,51,52,75,82,83,84].

For miR-21, amplification-based methods (qRT-PCR and dPCR) are the most widely used due to their high sensitivity, especially for circulating biomarkers. At the same time, biosensors and emerging optical methods offer promising prospects for rapid and non-invasive detection [16,17,18,54,55,56,57,58,59,60,69,70,71,72].

An essential aspect is the complementarity of the methods: tissue techniques provide specificity and morphological context, while liquid methods and biosensors allow for dynamic monitoring of the disease [7,42,43,51,52,54,55,56,57,58,59,60,69,70,71,72,73,74,75,77,82,83,84]. Thus, multiplatform integration of detection methods may lead to a more complete characterization of biomarkers and optimization of patient management. A global comparison of the main detection technologies used for HER2 and miR-21 biomarkers is presented in Table 3.

As highlighted in Table 3, there is a significant discrepancy between the analytical performance of emerging methods and their current level of clinical implementation, highlighting the need for further validation and standardization.

The evolution of detection methods is moving towards miniaturized, automated and integrated technologies, capable of providing rapid and accurate results in a clinical context. Portable biosensors, lab-on-a-chip platforms and artificial intelligence-based technologies have the potential to transform oncology diagnostics, facilitating personalized medicine [67,73,74].

For HER2, future directions include improving detection of “HER2-low” forms and integrating circulating biomarkers. In the case of miR-21, standardization of methods and clinical validation are essential for implementation in routine practice [13,79,82,83,84,86,87,88,89,90,91,92,93,94].

Overall, progress in detection methods will allow not only early diagnosis, but also continuous monitoring of the disease, contributing to the optimization of therapeutic strategies and improving the prognosis of breast cancer patients.

In this context, it becomes evident that traditional approaches based on individual biomarkers are insufficient to capture the complexity of tumor biology.

A key conceptual aspect highlighted by this review is the fundamental complementarity between HER2 and miR-21 biomarkers, which can be interpreted within an integrative static–dynamic model of tumor biology. While HER2 provides a point-in-time tissue characterization (“molecular snapshot”) used to guide targeted therapy, miR-21 reflects the systemic dynamics of the tumor, allowing real-time monitoring of progression and therapeutic response. This duality suggests that the integration of tissue and circulating biomarkers is not just a technological extension, but a conceptual shift in precision oncology, oriented towards a continuous and adaptive assessment of the disease.

**Figure 8:**
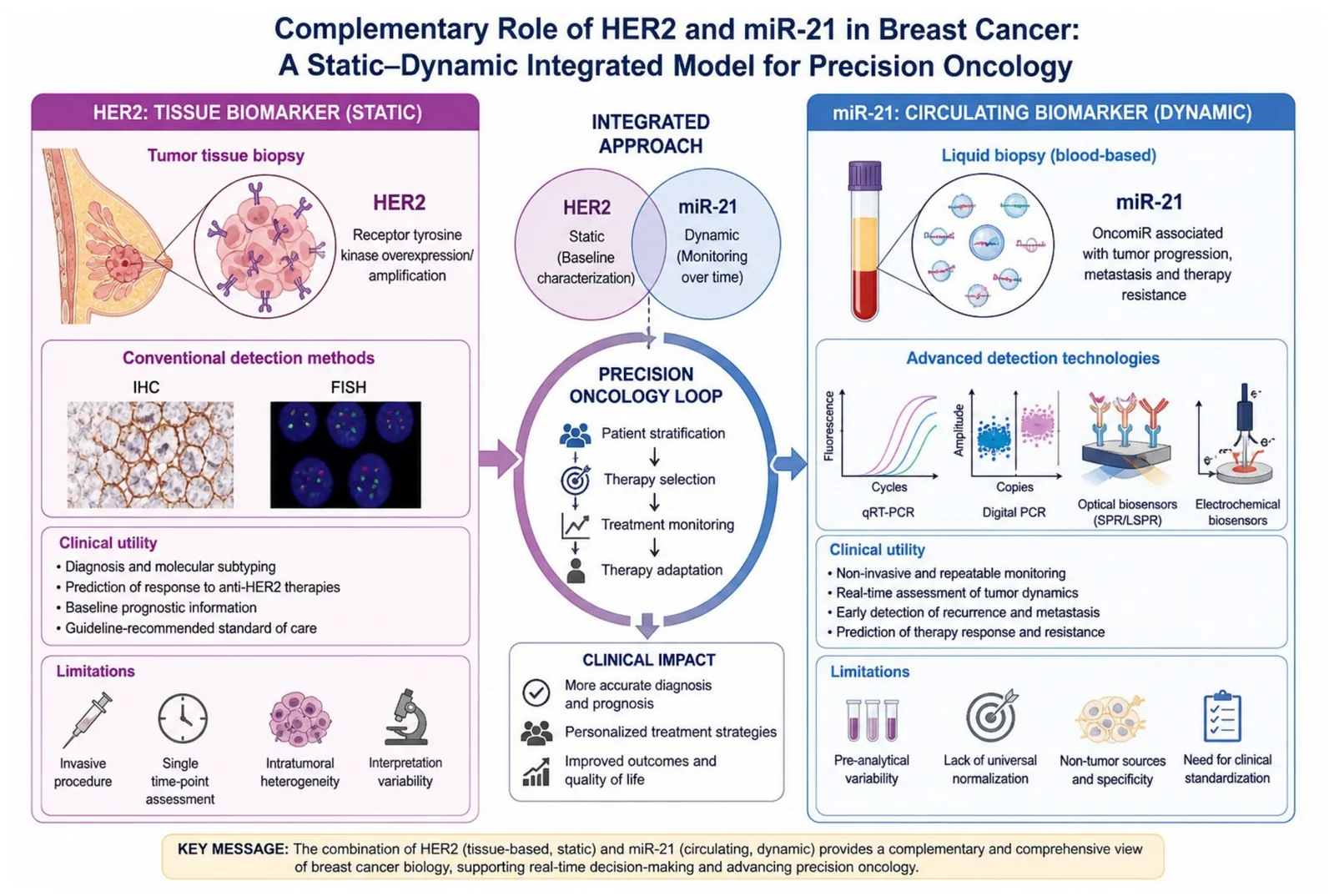
Static–dynamic integrative model of HER2 and miR-21 biomarkers in breast cancer.

The complementary concept of HER2 and miR-21 biomarkers is schematically illustrated in Figure 8, which proposes an integrative static–dynamic model of precision oncology. This model highlights the role of HER2 in initial tumor characterization and miR-21 in dynamic monitoring of disease progression, suggesting a continuous loop of therapeutic adaptation.

HER2, as a tissue biomarker, provides a point-in-time molecular characterization of the tumor (“snapshot”), being mainly used for patient stratification and selection of targeted therapies. In contrast, miR-21, as a circulating biomarker, reflects the systemic dynamics of the disease, allowing real-time monitoring of tumor progression and response to treatment. The integration of these two types of biomarkers is represented by a continuous loop of precision oncology, which includes patient stratification, therapy selection, response monitoring and treatment adaptation. The model highlights the complementarity between static and dynamic assessment, suggesting a multimodal approach to optimize breast cancer management [29,30,31,32,33,34,35,36,37,38,39,40,41,42,43,46,47,48,49,50,51,52,53,80,81].

### 4.4. Clinical Translation, Challenges and Perspectives

Current ASCO/CAP recommendations reaffirm validated HER2 testing by immunohistochemistry (IHC), with in situ hybridization (ISH) used according to the established testing algorithm, while emphasizing standardized interpretation and reporting of HER2 results, including the distinction between IHC 0 and IHC 1+ [68]. Emerging electrochemical and optical biosensors may offer advantages in analytical sensitivity, speed, miniaturization, and point-of-care applicability; however, they currently remain complementary technologies because standardization, reproducibility, and clinical validation in relevant patient cohorts are not yet comparable with established HER2 assays [76].

Until large concordance studies vs. IHC/FISH (*n* > 300, ASCO/CAP concordance ≥ 95% requirement) and CLIA validation are completed, biosensors remain investigational and must not be presented as guideline-equivalent. Hence, emerging electrochemical biosensors are not intended to replace IHC/FISH for tissue subtyping, but to complement/replace serum HER2 ELISA.

#### 4.4.1. Predictive Value of ncRNAs for Therapy Response: Beyond Diagnosis

While the primary focus of this review is the development of diagnostic biosensors, the potential clinical value of miR-21 extends beyond diagnosis. Increasing evidence suggests that miR-21 may also provide information relevant to therapeutic response and resistance, particularly in HER2-targeted therapy and chemotherapy. However, the level of evidence remains heterogeneous [44], and clinical validation as a treatment-selection biomarker is still limited, as presented in Table 4.

Overall, the available evidence supports a potential association between elevated miR-21 and treatment resistance, particularly for trastuzumab-based therapy and chemotherapy. However, most clinical studies remain observational or exploratory, while direct prospective validation of miR-21 as a predictive biomarker for treatment selection is lacking. Evidence regarding antibody–drug conjugates and immune checkpoint inhibitors is currently more limited and should therefore be considered hypothesis-generating. This evidence gap is particularly relevant for biosensor development, as analytical detection of miR-21 has advanced considerably faster than its clinical validation as a therapy-response biomarker.

#### 4.4.2. Impact of Tumor Microenvironment Heterogeneity on ncRNA Detection Accuracy and Reproducibility

Tumor microenvironment (TME) heterogeneity represents an important but insufficiently addressed source of variability in circulating miR-21 measurements. In particular, hypoxia, stromal and immune-cell contributions, the distribution of miR-21 between free and extracellular-vesicle-associated fractions, and pre-analytical factors such as hemolysis may alter the measured circulating concentration independently of tumor burden. These factors are therefore relevant when translating analytically sensitive biosensors into clinically heterogeneous samples (Table 5).

Collectively, these factors indicate that analytical sensitivity alone may not guarantee clinically reliable miR-21 quantification. A biosensor may achieve an excellent analytical LOD under controlled conditions but show substantially different performance in heterogeneous clinical samples if the cellular origin, extracellular-vesicle fraction, or pre-analytical conditions are not standardized. Future studies should therefore incorporate TME-aware normalization and standardized pre-analytical procedures, including reporting of hemolysis and sample-processing conditions. Where extracellular-vesicle-associated miR-21 is specifically investigated, isolation and characterization procedures should also be clearly reported.

#### 4.4.3. ncRNA-Related Clinical Trials and Recent Translational Studies in Breast Cancer

To place the growing body of ncRNA research in a clinically relevant context, we also reviewed registered clinical studies and recent translational investigations addressing ncRNA biomarkers in breast cancer. These studies cover several potential applications, including diagnosis, monitoring of treatment response, assessment of therapeutic sensitivity, and investigation of mechanisms associated with treatment resistance. Table 6 summarizes the main registered studies and recent translational reports, highlighting the biomarker investigated, patient population, study design, recruitment status, and available publication data.

Overall, the available evidence indicates that ncRNA biomarkers, particularly miRNAs, are progressively moving from analytical and exploratory research toward clinical investigation. However, the evidence remains heterogeneous, with several studies still recruiting or lacking published clinical outcomes, while recent translational studies remain predominantly preclinical. Thus, although circulating and exosomal ncRNAs show potential for diagnosis, treatment monitoring, and resistance assessment, their incorporation into routine breast cancer management will require prospective validation in larger, well-characterized cohorts and harmonization of analytical and pre-analytical procedures.

This gap between promising biomarker performance and demonstrated clinical utility further supports the need for standardized validation and prospective evaluation before ncRNA-based biosensors can be considered for routine clinical decision-making.

#### 4.4.4. Clinical Challenges, Methodological Limitations and Perspectives for Translational Integration

Despite significant advances in the field of cancer biomarkers, the effective integration of emerging biomarkers, such as miR-21, into routine clinical practice remains limited by a number of critical factors. One of the most important obstacles is the lack of standardization of preanalytical and analytical protocols, including variability in biological sample collection, processing, and storage, as well as the absence of universal methods for normalizing microRNA expression [59,60,87,90,91]. These limitations significantly affect the reproducibility of results and comparability between studies, preventing large-scale clinical validation.

In addition, the specificity of miR-21 as a tumor biomarker is influenced by the fact that it is not exclusively associated with breast cancer, being overexpressed in other types of neoplasia or inflammatory processes, which may generate false positive results in the context of liquid biopsy [56,57,77].

Also, interindividual biological heterogeneity and the contribution of non-tumor sources to circulating microRNA levels are additional factors complicating data interpretation.

Regarding HER2, although it is a well-validated and clinically standardized biomarker, there are emerging challenges related to intra-tumoral heterogeneity and the redefinition of molecular subtypes, especially in the context of the “HER2-low” category [57,58,77,102,103]. This intermediate category poses diagnostic challenges in terms of detection thresholds and interpretation of immunohistochemical results, with direct implications for patient eligibility for targeted therapies.

Unlike HER2, which benefits from over 20 years of clinical standardization, miR-21 detection technologies remain in early translational stages. To provide clarity, we stratified technologies according to Technology Readiness Levels (TRL) in Table 7. HER2 testing is at TRL 9 (commercial clinical use): Immunohistochemistry (IHC, ASCO/CAP 2023 guidelines), fluorescence in situ hybridization (FISH) for equivocal cases, and serum HER2 ECD ELISA are FDA-cleared and integrated into breast cancer staging and therapy selection. Emerging HER2 electrochemical biosensors discussed here are positioned as potential point-of-care alternatives to ELISA, but are themselves at TRL 4–5 (clinical validation in small patient cohorts, *n* = 20–60).

The miR-21 biosensors are at TRL 3–5 (proof-of-concept to clinical validation): TRL 3—Laboratory proof-of-concept in which most studies (68% of miR-21 articles included) used synthetic miR-21 in buffer or spiked serum, demonstrating analytical sensitivity without patient samples. These include FET-based, plasmonic and many nanocomposite electrochemical sensors. TRL 4—Preclinical validation, including studies using breast cancer cell lines (MCF-7, SK-BR-3) or exosomes, showing discrimination of cancer vs. normal cells, but without a clinical cohort.

TRL 5—Clinical validation: only 32% of diagnostic studies tested miR-21 in patient serum/plasma (*n* = 20–150) against qRT-PCR as a reference standard. Even in this group, sample size was limited (<100 in 75%), blinding was often unclear, and no study performed multicenter validation or followed REMARK/miR QC guidelines. There are no TRLs 6–9. To date, no miR-21 biosensor has reached analytical validation. Commercially available miR-21 assays are limited to laboratory qRT-PCR panels (e.g., Qiagen miRCURY, Thermo Fisher TaqMan), which require RNA extraction and are not point-of-care. HER2 biosensors are promising emerging technologies with potential for future clinical implementation. However, their translation into routine practice will require further analytical standardization, clinical validation, and demonstration of clinical utility. In contrast, miR-21 biosensors still require large-scale prospective validation before clinical implementation can be considered.

To compare the translational maturity of the reviewed technologies, the approaches in Table 7 were positioned according to their reported stage of development and validation, considering the experimental setting, sample type, extent of clinical evaluation, and current clinical implementation. Lower readiness levels were assigned to proof-of-concept or laboratory-based technologies, whereas approaches supported by patient-derived samples, clinical validation, or established clinical use were assigned progressively higher levels. This classification is intended as a comparative framework and does not represent a formal regulatory TRL assessment of individual studies.

This distinction is now summarized in Table 7 to prevent overinterpretation of clinical readiness.

Future clinical implementation will depend on further analytical standardization, validation in larger prospective clinical cohorts, assessment of inter-laboratory reproducibility, regulatory evaluation, and demonstration of clinical utility.

#### 4.4.5. Clinical Decision-Making Framework: From Biomarker to Bedside

**Figure 9:**
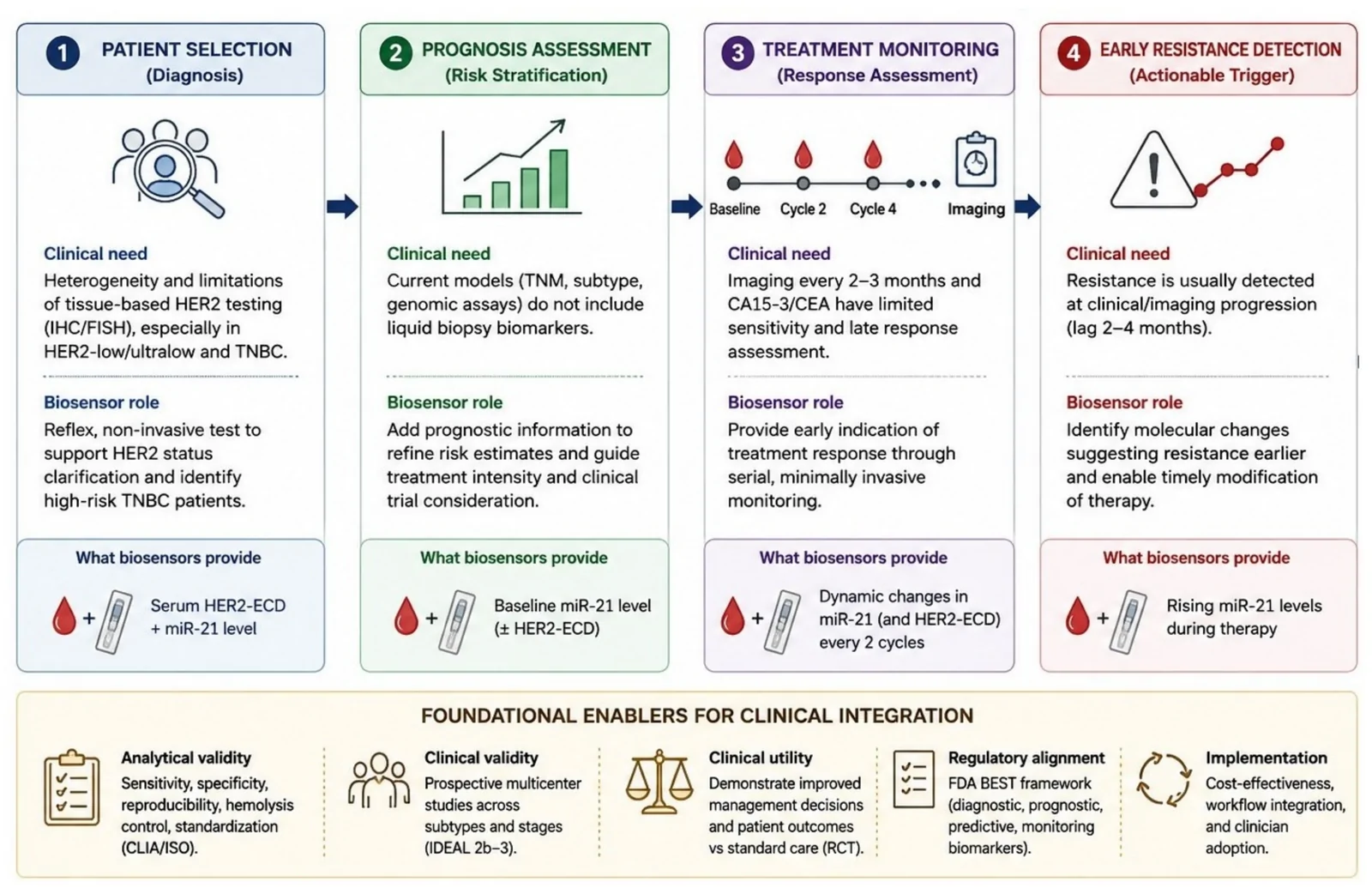
Integration of HER2/miR-21 biosensors into clinical decision making.

The potential integration of HER2 and miR-21 biosensors into clinical practice should be considered across the entire clinical decision-making pathway, rather than solely in terms of analytical performance. As summarized in Figure 9, emerging biosensor technologies could provide complementary information at four clinically relevant decision points: patient selection at diagnosis, prognostic assessment, longitudinal treatment monitoring, and early detection of therapeutic resistance. Their potential contribution differs according to the clinical question and the maturity of the available evidence [104,105].

At present, these applications should be regarded as complementary rather than as replacements for established diagnostic and clinical assessment methods. HER2 biosensors have the closest path toward clinical implementation because HER2 is already an established biomarker with standardized diagnostic and therapeutic frameworks, whereas miR-21 remains investigational and requires greater analytical and clinical standardization. In particular, prospective multicenter validation, reproducible pre-analytical procedures, clinically validated cut-offs, and demonstration of clinical utility are required before biosensor-derived measurements can be used to guide treatment decisions. Thus, the principal near-term value of HER2/miR-21 biosensors may lie in providing rapid and minimally invasive molecular information that may complement conventional pathology, imaging, and clinical assessment and support personalized clinical decision-making [68,104,105].

Another critical aspect is the integration of data from multiple biomarkers. Although the combined use of HER2 and miR-21 provides a more comprehensive view of tumor biology, the lack of standardized data integration platforms and robust predictive models limits the clinical applicability of this approach [63,72,75,78,85]. In this context, the development of multimodal strategies that combine tissue and circulating biomarkers, along with advanced analytical methods and artificial intelligence algorithms, represents a promising direction for precision oncology. Considering these aspects, overcoming the listed challenges requires concerted efforts towards methodological standardization, rigorous clinical validation and the development of integrated analysis platforms, capable of transforming molecular biomarkers from research tools into essential components of current clinical practice.

## 5. Conclusions

In the context of precision oncology, the identification and characterization of relevant molecular biomarkers is an essential component for optimizing diagnosis, prognosis and therapeutic strategies. This review highlights the complementary role of HER2 and miR-21 biomarkers in breast cancer, their fundamental differences, and also their synergistic potential in clinical practice and translational research. Bibliometric analyses revealed the intellectual structure, research trends, and collaboration patterns within the field of HER2 and miR-21 detection in breast cancer. Particularly, the keyword co-occurrence network revealed a highly interconnected conceptual landscape dominated by three major research pillars: breast cancer biomarkers (particularly HER2 and miR-21), biosensor technologies, and nanomaterial-assisted signal amplification strategies.

Overall, the collaboration network demonstrates that research on biosensors for HER2/ERBB2 and miR-21 detection in breast cancer has evolved into a highly internationalized and multidisciplinary scientific domain. A major bibliometric insight is the evident shift in high publication output toward Asia, particularly China, India, and Iran, which also show extensive collaborative connectivity within the analyzed network. Simultaneously, the United States and leading European countries continue to play indispensable roles in technological validation, interdisciplinary integration, and clinical translation. These findings underscore the increasingly collaborative nature of innovation in biosensor-driven oncology diagnostics and reflect the global strategic importance of developing sensitive, selective, and clinically applicable biosensing platforms for precision cancer management.

HER2 remains an established biomarker with well-defined predictive value, integrated into clinical guidelines and essential for the selection of targeted therapies. Standard methods of determination, such as immunohistochemistry and in situ hybridization, provide robust and validated results, but are limited by their invasive nature and the lack of continuous dynamic monitoring. In contrast, miR-21 is an emerging biomarker with prognostic value and significant potential for non-invasive disease monitoring, being detectable in biological fluids and reflecting tumor dynamics in real time. However, miR-21 expression and biological function may differ among tumors with distinct genetic backgrounds, so miR-21 utility must be interpreted in a subtype-specific context.

Comparative analysis of detection methods highlights that, although conventional techniques remain the clinical standard, they are increasingly complemented by advanced technologies, such as digital PCR, optical and electrochemical biosensors. These emerging methods offer significant advantages in terms of sensitivity, rapidity and potential for miniaturization, but their clinical implementation is still limited by the lack of standardization and large-scale validation.

Regarding future perspectives, a key aspect highlighted in this review is the need for an integrated approach, combining biomarkers of different biological natures and complementary analytical methods. The concomitant use of HER2 (a tissue-based, predictive biomarker) and miR-21 (a circulating, prognostic, and dynamic biomarker) may provide a more complete characterization of the disease, contributing to more accurate patient stratification and real-time treatment adaptation.

Looking ahead, major directions include standardization of microRNA detection methods, clinical validation of biosensors, and integration of emerging technologies into routine diagnostic workflows. In addition, the development of multimodal platforms and point-of-care systems, as well as the integration of molecular data with artificial intelligence algorithms, has the potential to significantly transform breast cancer management.

In conclusion, progress in the field of HER2 and miR-21 biomarkers and associated detection methods supports the transition to personalized medicine, based on complex molecular characterization of tumors. The integration of these biomarkers into multimodal diagnostic and therapeutic strategies represents a promising direction for improving the prognosis and quality of life of breast cancer patients.

## Figures and Tables

**Table 1:** Top 10 most-cited papers in the period 2016–2026.

First Author/Year	Article Title	Source Title	IF/2025	Citations	References
Wang, Y.S. (2024)	Bimetallic Single-Atom Nanozyme-Based Electrochemical-Photothermal Dual-Function Portable Immunoassay with Smartphone Imaging	ANALYTICAL CHEMISTRY	1.7	200	[19]
Zubair, M (2021)	Advanced Approaches to Breast Cancer Classification and Diagnosis	FRONTIERS IN PHARMACOLOGY	5.4	191	[20]
Zhu, Dan (2017)	Label-Free Electrochemical Sensing Platform for MicroRNA-21 Detection Using Thionine and Gold Nanoparticles Co-Functionalized MoS2 Nanosheet	ACS APPLIED MATERIALS & INTERFACES	7.8	168	[21]
Rafiee-Pour, HA (2016)	A novel label-free electrochemical miRNA biosensor using methylene blue as redox indicator: application to breast cancer biomarker miRNA-21	BIOSENSORS & BIOELECTRONICS	11.8	162	[22]
Zhang, J (2018)	A ratiometric electrochemical biosensor for exosomal microRNA detection based on bipedal DNA walkers propelled by locked nucleic acid modified toehold mediate strand displacement reaction	BIOSENSORS & BIOELECTRONICS	11.8	149	[23]
Ali, MA (2017)	Microfluidic Immuno-Biochip for Detection of Breast Cancer Biomarkers Using Hierarchical Composite of Porous Graphene and Titanium Dioxide Nanofibers	ACS APPLIED MATERIALS & INTERFACES	7.8	141	[24]
Su, XM (2023)	Integrated SERS-Vertical Flow Biosensor Enabling Multiplexed Quantitative Profiling of Serological Exosomal Proteins in Patients for Accurate Breast Cancer Subtyping	ACS NANO	17.3	139	[25]
Saeed, AA (2017)	DNA biosensors based on gold nanoparticles-modified graphene oxide for the detection of breast cancer biomarkers for early diagnosis	BIOELECTROCHEMISTRY	4.9	136	[26]
Arya, SK (2018)	Capacitive aptasensor based on interdigitated electrode for breast cancer detection in undiluted human serum	BIOSENSORS & BIOELECTRONICS	11.8	133	[27]
Tian, L (2018)	Gold nanoparticle superlattice assembly for electrochemical biosensor detection of microRNA-21	BIOSENSORS & BIOELECTRONICS	11.8	132	[28]

**Table 2:** Comprehensive comparison of HER2 vs. miR-21 as breast cancer biomarkers.

Parameter	HER2	miR-21	References
Biomarker type	Receptor protein (HER2/ERBB2)	MicroRNA (onco-miR)	[3,4,5,29,30,31,32,33,38,41]
Localization	Cell membrane; transmembrane receptor	Intracellular and circulating; detectable in plasma/serum and exosomes	[3,4,5,14,15,16,17,18,43,50,51]
Biological role	Membrane receptor tyrosine kinase; activates HER2-associated oncogenic signaling	Post-transcriptional regulator; targets tumor-suppressor pathways including PTEN, PDCD4 and SPRY2	[29,30,31,32,34,35,36]
Primary function	Cell proliferation, survival and tumor progression through HER2 signaling	Promotion of proliferation, invasion, metastasis and treatment resistance	[29,30,31,32,34,35,36,41,44]
Clinical value	Predictive and prognostic; directly relevant to treatment selection	Predominantly prognostic and potentially useful for disease monitoring	[7,8,13,14,15,16,17,18,41,44,45]
Sample type	Tumor tissue; serum/plasma for soluble HER2 ECD	Tissue, serum/plasma; free or exosomal circulating miR-21	[17,18,43,46,51,53]
Clinical use	Standard of care; HER2 status is essential for treatment selection	Under clinical validation; not routinely incorporated into clinical guidelines	[29,30,31,32,33,36,37,38,41,42,47,48]
Dynamic monitoring	Limited; tissue assessment provides a snapshot at biopsy	Potentially excellent; non-invasive repeated assessment in biological fluids	[7,15,16,17,18,52,54,55]
Therapeutic target	Yes; HER2-targeted therapy is established	Indirect; no established miR-21-directed therapy-selection role	[17,18,43,50,51]
Detection gold standard	IHC as first-line clinical assessment; FISH for ERBB2 amplification/equivocal cases	qRT-PCR is widely used; dPCR provides highly sensitive absolute quantification	[7,42,43,54,55,56]
Emerging detection (this review)	Electrochemical immunosensors/aptasensors, FET and plasmonic platforms	Electrochemical genosensors/aptasensors, FET, SPR/LSPR, SERS and amplification-assisted approaches	[19,21,22,23,24,25,26,27,28,57,58,59,60,61,62,63,64,65,66,67,68,69,70,71,72,73,74,75]
LOD/analytical sensitivity	Electrochemical and optical platforms can reach very low concentration ranges; performance is platform-dependent	Very high analytical sensitivity reported for dPCR and emerging biosensors; performance is platform-dependent	[44,45,58,59,60,61,62,63,64,65,66,67,76]
Diagnostic performance	Conventional IHC/FISH methods have extensive clinical validation and high specificity	qRT-PCR/dPCR provide high analytical sensitivity, but clinical diagnostic validation remains less mature	[7,52,54,55,57,77]
Prognostic value	Established prognostic relevance, particularly in HER2-positive disease	Elevated miR-21 is associated with advanced disease, metastasis, treatment resistance and poorer survival	[13,30,31,32,33,42,47,48]
Predictive value	Established; HER2 status guides selection of anti-HER2 therapy	Not established for routine therapy selection; potential role in response/resistance monitoring	[7,8,42,47,48,49]
Guideline status	Integrated into clinical guidelines and routine breast cancer management	No routine guideline role; investigational/clinical-validation stage	[7,15,16,17,18,43,51,52,54,55]
Commercial assay	Established clinical HER2 testing platforms are available	No routine FDA-cleared point-of-care miR-21 biosensor identified in the review	[7,52,54,56,57,68,77,78,79]
Strengths	Standardized clinical use, validated methods, direct therapeutic relevance	Non-invasive sampling, potential for repeated monitoring, high analytical sensitivity	[7,14,15,16,17,18,43,51,52,54,55,56]
Limitations	Invasive tissue sampling, tumor heterogeneity, limited ability for continuous monitoring	Biological/pre-analytical variability, normalization issues, lack of standardization and limited large-scale clinical validation	[43,46,51,52,53,54,55,56,57,77]

**Table 3:** Comparing the main detection technologies and methods used for HER2 and miR-21 biomarkers in breast cancer.

Technology	Biomarker	LOD/Analytical Sensitivity	Specificity/Reproducibility	Clinical Validation	Turnaround/Cost	Regulatory Status	Main Barrier	References
IHC	HER2	Semi-quantitative (0–3+) scoring	Inter-observer variability; non-standardized scoring	Clinical standard; guideline-based	1–2 days/relatively low	Established clinical use	Pre-analytical and interpretative variability	[7,42,43,51,52]
FISH/CISH	HER2	Gene amplification assessment	High specificity; affected by tumor heterogeneity	Reference/confirmatory method	2–3 days/higher cost	Established clinical use	Specialized equipment and expertise	[7,42,43,51,52]
qRT-PCR	miR-21	High analytical sensitivity; relative, ratio-dependent quantification	Normalization-related; reagent-dependent	Widely used in research; clinical standardization incomplete	Hours/relatively low	Research/LDT use	Normalization and pre-analytical variability	[54,55]
dPCR	miR-21	Very high sensitivity; absolute quantification	High analytical precision	Advanced molecular testing; limited miR-21 clinical implementation	Hours/higher cost	Emerging	Cost and technical complexity	[56]
ELISA (HER2)	HER2	ng/mL range	Moderate	Used for serum HER2 monitoring	~3 h/relatively low	Clinical use	Lower sensitivity; no tissue localization	[82,83,84]
SPR/LSPR	HER2/miR-21	Very high sensitivity	Label-free; high analytical sensitivity	Preclinical/proof-of-concept	Minutes/high R&D cost	Experimental	Complexity and limited clinical validation	[57,58,77]
Electrochemical aptasensor	HER2/miR-21	fM range; reported	High analytical sensitivity; nanomaterial-dependent	Mainly laboratory validation	<1 h/potentially low per test	Experimental	Matrix interference; limited large-scale validation	[19,21,22,23,24,25,26,27,28,62,63,64,65,66,68,72,75]
NGS	HER2/miR-21	High-throughput molecular profiling; assay-dependent sensitivity; no single concentration-based LOD	High analytical specificity with validated sequencing and bioinformatic workflows; reproducibility depends on assay standardization	Established for selected molecular oncology applications; limited routine use for miR-21 detection	Days/high	Established for selected oncology applications	Cost, turnaround time, complex data analysis, and limited standardization for routine miRNA testing	[12,13]

**Table 4:** Evidence supporting the potential predictive value of miR-21 for therapeutic response.

Therapy	Association with High miR-21	Level of Evidence	Mechanism	Clinical Cohort Data	References
Trastuzumab/Pertuzumab	High miR-21 associated with resistance/poorer response	Clinical + preclinical	PTEN and PDCD4 suppression → PI3K/AKT signaling and survival pathways	HER2+ neoadjuvant cohort (*n* = 127): circulating miR-21 associated with overall survival; no significant association with pCR reported	[44,45]
T-DM1/T-DXd	Potential association with emerging resistance; evidence insufficient	Mainly preclinical/indirect	PTEN/PI3K/AKT and resistance-related signaling pathways	No validated clinical miR-21 predictive dataset identified	[95]
Chemotherapy (taxanes/anthracyclines)	Higher miR-21 associated with poorer treatment response in some cohorts	Clinical + preclinical	Suppression of tumor-suppressive pathways, including PTEN and PDCD4, favoring survival and reduced apoptosis	Prospective NACT data support an association between circulating miR-21 and treatment response; exact AUC 0.81 should not be attributed to miR-21	[36,37,86,87]
Immunotherapy (anti-PD-1/anti-PD-L1)	Potential association with immune resistance; not clinically established	Preclinical/hypothesis-generating	miR-21 influences macrophage polarization and immune signaling; depletion can enhance PD-1 blockade in experimental models	No robust clinical HR = 1.74 for miR-21 in IMpassion130 identified	[96]

**Table 5:** TME factors affecting miR-21 detection reproducibility.

TME Factor	Effect on Circulating miR-21	Reported Effect	Impact on Biosensor Accuracy	References
Hypoxia (HIF-1α)	↑ transcription + exosome release	3–5× ↑	Overestimation of tumor burden	[97,98]
CAF content > 50%	CAF exosomal miR-21 secretion	miR-21 enriched in CAF-derived exosomes	Signal may reflect stromal contribution, not only tumor- cell burden	[99]
TAM M2 infiltration	miR-21 contributes to macrophage polarization and tumor-associated immunosuppression	Mechanistic evidence; no validated 2–3× clinical increase established	Circulating miR-21 may reflect immune-cell/TME contribution in addition to tumor-cell expression	[96]
Free vs. exosomal miRNA	Circulating miRNAs occur in both vesicle-associated and non-vesicular Ago2-bound fractions	The relative distribution is miRNA-dependent; circulating miRNAs associated with Ago2 rather than extracellular vesicles	Biosensors measuring total circulating miR-21 may detect both molecular fractions, which may have different stability and biological significance and can therefore contribute to variability in the measured signal	[100,101]
Hemolysis/blood cell contamination	Release of blood-cell-associated miRNAs and distortion of circulating miRNA profiles; miR-21 may also be affected at high levels of hemolysis	Hemolysis-dependent; magnitude varies by miRNA and degree of contamination	Can produce falsely elevated or distorted biomarker signals and compromise normalization	[88,89]

**Table 6:** Summary of ncRNA-related clinical trials and recent translational studies in breast cancer.

ncRNA Target/Biomarker	Breast Cancer Subtype/Population	Trial Phase/Study Design	Recruitment Status	Estimated Completion/Publication	References/NCT
miR-21 + miR-34a panel	Breast cancer; Luminal A/B, HER2+, basal-like	Observational clinical study	Completed	Published 2022	[86]
miR-21 (monitoring)	Early-stage breast cancer, Stage 0–III	Interventional; Phase II trial	Recruiting	Est. study completion Nov 2028	[90]
Circulating miRNA panel: let-7a, miR-21, miR-145, miR-155, miR-195	Breast cancer receiving neoadjuvant chemotherapy	Prospective multicenter observational cohort	Completed	Published 2022–2023	[87,91]
miR-373 + miR-425-5p	Early breast cancer vs. controls	Observational diagnostic case–control study	Status requires registry verification	Registry completion estimate previously 2023	[102]
Circulating miRNA profile for chemoresistance	Triple-negative breast cancer (TNBC)	Phase II; single-arm	Recruiting	Primary completion Mar 2027; study completion Aug 2030	[103]
UPK1A-AS1 + UNC5B-AS1 lncRNA panel	Breast cancer	Observational	Recruiting/registry requires update	Registry estimate previously Dec 2025	[92]
miR-21 (before/after neoadjuvant therapy)	Invasive breast cancer, Stage IIB–IIIC; all subtypes	Observational prospective cohort	Unknown; last known not yet recruiting	Estimated completion Jan 2024	[93]
Circulating miRNA panel for hormone sensitivity	Metastatic or locally advanced hormone-dependent breast cancer	Phase IV; interventional, single-arm	Completed	Completed Jan 2016	[94]
circRNA crVDAC3	HER2-low breast cancer; T-DXd resistance	Preclinical/PDX translational study	Published	Published 2024	[95]
Exosomal miR-205/Rab27A-associated sEV miRNAs	DCIS → invasive breast cancer progression	Translational/preclinical study	Published	Published 2025	[101]

**Table 7:** Maturity classification of HER2 and miR-21 detection technologies.

Technology Category	Representative Platforms	TRL	Validation Level	Clinical Status	References
HER2—Gold Standard	IHC (HercepTest), FISH (PathVysion)	TRL 9	10,000 patients, guidelines	FDA-cleared, routine use, ASCO/CAP	[7,42,43,51,52,79,104]
HER2—Serum ELISA	Siemens, R&D Systems HER2 ECD	TRL 9	Clinical cohorts	FDA-cleared, monitoring	[82,83,84]
HER2—Emerging biosensors	Electrochemical immunosensors in this review	TRL 4–5	20–60 patient sera vs. ELISA	Experimental, no commercial kit	[19,24,25,26,27,28,68]
miR-21—qRT-PCR	Qiagen miRCURY, TaqMan	TRL 7	Clinical research use	Commercial RUO, not diagnostic	[54,55,56]
miR-21—Electrochemical biosensors	AuNPs/graphene, MXene, 78% of included studies	TRL 3–4	Mostly buffer/spiked serum	Lab proof-of-concept	[21,22,23,26,27,28,62,63,64,65,68,72,75]
miR-21—Advanced transduction	FET, SPR, SERS, plasmonic	TRL 3	Cell lines, exosomes	Experimental phase	[57,58,59,60,77]
miR-21—Clinical biosensors	8/25 studies with patient serum (this review)	TRL 5	20–150 patients vs. qRT-PCR	Preclinical clinical validation only	[21,22,23,24,25,26,27,28,68]

Legend. TRL: 1 = basic principle, 3 = proof-of-concept, 5 = clinical validation small cohort, 9 = commercial clinical use.

## Data Availability

No new data were created or analyzed in this study. Data sharing is not applicable to this article.

## Supplementary Materials

The following supporting information can be downloaded at: https://www.mdpi.com/article/10.3390/diagnostics16193179/s1, Supplementary Material S1: PRISMA 2020 checklist; Supplementary Material S2: Risk of bias.
