# Supplementary material for: Detection Strategies for HER2 and miR-21 in Breast Cancer: Current Approaches and Future Perspectives- Hybrid Systematic Review and Bibliometric Mapping
Source: Diagnostics (Basel). 2026 Sep 30;16(19):3179. doi: 10.3390/diagnostics16193179 (PMC13664366; doi:10.3390/diagnostics16193179)
Supplement: Supplementary file 1 [file diagnostics-16-03179-s001.zip › S2_Risk of bias.pdf]

Supplementary material S2

To provide a structured overview of methodological considerations across the included evidence base, the 305 studies were assessed using a predefined five-domain framework. The assessment considered sample selection and clinical relevance, endogenous target relevance, analytical validation, use of an independent comparator or reference method, and clinical applicability or discrimination. Given the heterogeneous nature of the included studies, ranging from early-stage proof-of-concept investigations to studies involving clinical samples, the framework was designed to distinguish explicit methodological concerns from limitations related primarily to the developmental or translational stage of the technology. Accordingly, the absence of information in the available study records was classified as unclear rather than automatically interpreted as a high methodological concern.

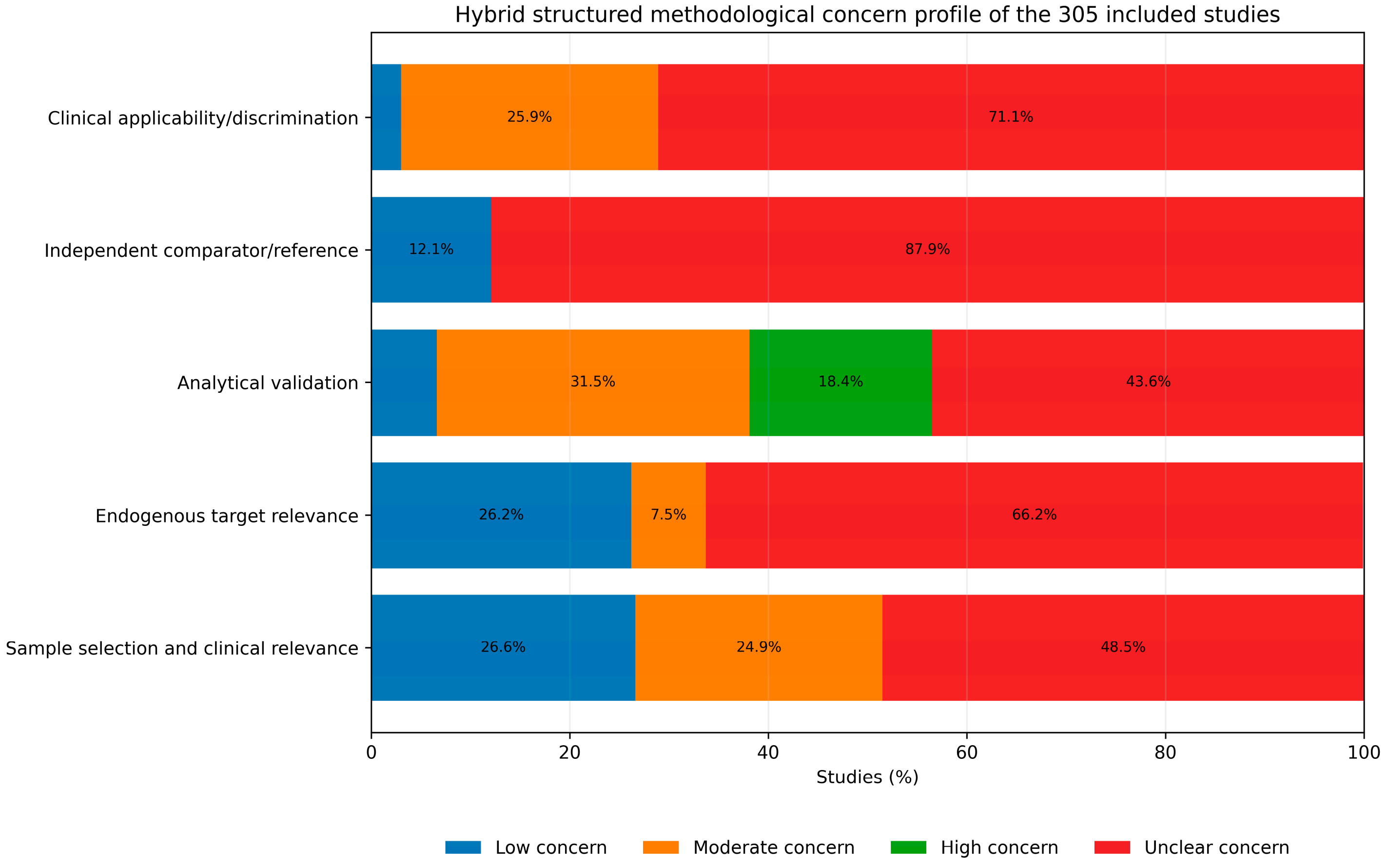

Figure S1. Structured methodological concern profile of the 305 studies included in the review. Studies were evaluated across five domains: sample selection and clinical relevance, endogenous target relevance, analytical validation, independent comparator/reference use, and clinical applicability/discrimination. Each domain was classified as low, moderate, high, or unclear concern according to predefined criteria. This framework provides a structured characterization of methodological and translational evidence and does not represent a formal risk-of-bias assessment using a validated instrument.

Overall, the analysis revealed substantial heterogeneity in the methodological and translational maturity of the included studies. High methodological concern was identified only when explicit limitations could be established according to the predefined criteria, whereas insufficiently reported information was retained in the unclear category. This approach avoids interpreting early-stage proof-of-concept studies

or incomplete reporting as direct evidence of poor methodological quality. The resulting profile highlights the progressive transition from analytical feasibility studies toward clinically relevant biosensing applications and emphasizes the need for more comprehensive analytical validation, independent comparison with established reference methods, and evaluation in well-characterized clinical cohorts.
